# Unisexual and Heterosexual Meiotic Reproduction Generate Aneuploidy and Phenotypic Diversity *De Novo* in the Yeast *Cryptococcus neoformans*

**DOI:** 10.1371/journal.pbio.1001653

**Published:** 2013-09-10

**Authors:** Min Ni, Marianna Feretzaki, Wenjun Li, Anna Floyd-Averette, Piotr Mieczkowski, Fred S. Dietrich, Joseph Heitman

**Affiliations:** 1 Department of Molecular Genetics and Microbiology, Duke University Medical Center, Durham, North Carolina, United States of America; 2 Department of Genetics, School of Medicine, University of North Carolina, Chapel Hill, North Carolina, United States of America; Carnegie Mellon University, United States of America

## Abstract

Aneuploidy is known to be deleterious and underlies several common human diseases, including cancer and genetic disorders such as trisomy 21 in Down's syndrome. In contrast, aneuploidy can also be advantageous and in fungi confers antifungal drug resistance and enables rapid adaptive evolution. We report here that sexual reproduction generates phenotypic and genotypic diversity in the human pathogenic yeast *Cryptococcus neoformans*, which is globally distributed and commonly infects individuals with compromised immunity, such as HIV/AIDS patients, causing life-threatening meningoencephalitis. *C. neoformans* has a defined **a**-α opposite sexual cycle; however, >99% of isolates are of the α mating type. Interestingly, α cells can undergo α-α unisexual reproduction, even involving genotypically identical cells. A central question is: Why would cells mate with themselves given that sex is costly and typically serves to admix preexisting genetic diversity from genetically divergent parents? In this study, we demonstrate that α-α unisexual reproduction frequently generates phenotypic diversity, and the majority of these variant progeny are aneuploid. Aneuploidy is responsible for the observed phenotypic changes, as chromosome loss restoring euploidy results in a wild-type phenotype. Other genetic changes, including diploidization, chromosome length polymorphisms, SNPs, and indels, were also generated. Phenotypic/genotypic changes were not observed following asexual mitotic reproduction. Aneuploidy was also detected in progeny from **a**-α opposite-sex congenic mating; thus, both homothallic and heterothallic sexual reproduction can generate phenotypic diversity *de novo*. Our study suggests that the ability to undergo unisexual reproduction may be an evolutionary strategy for eukaryotic microbial pathogens, enabling *de novo* genotypic and phenotypic plasticity and facilitating rapid adaptation to novel environments.

## Abstract 2

Unisexual and heterosexual reproduction in the pathogenic yeast *Cryptococcus neoformans* enables *de novo* phenotypic and genotypic plasticity with frequent aneuploidy and rapid adaptation.

## Author Summary

Aneuploidy refers to increases or decreases in the copy number of individual chromosomes (rather than of the entire haploid or diploid genome). In humans, aneuploidy is well known to be deleterious, causing genetic disorders such as Down syndrome (trisomy 21), and frequently occurring during mitosis in the genesis of cancer. By contrast, aneuploidy in fungi can be advantageous, conferring antifungal drug resistance and enabling rapid adaptive evolution. *Cryptococcus neoformans* is a globally distributed human pathogen that often infects patients with compromised immunity. It accounts for significant morbidity and mortality associated with HIV/AIDS and is linked to more than one million infections and >600,000 deaths per year world-wide. Although *C. neoformans* has a defined heterosexual cycle involving **a** and α cells, more than 99% of clinical and environmental isolates are α. Interestingly, *C. neoformans* α cells undergo α-α unisexual reproduction to generate diploid intermediates and infectious haploid spores. Sex is costly, though, and the question therefore arises as to why *C. neoformans* would undergo selfing unisexual, meiotic reproduction as opposed to more efficient asexual, mitotic reproduction. We show here that unisexual, meiotic reproduction *in C. neoformans* results in aneuploidy, creating advantageous genetic diversity *de novo*.

## Introduction

Aneuploidy, a condition in which cells have an abnormal number of chromosomes, can cause deleterious effects in organisms throughout the eukaryotic tree of life. Aneuploidy underlies several common human genetic diseases, including trisomy 21 in Down syndrome and trisomy 13 in Patau syndrome [1],[2], and is detected in more than 90% of solid tumors [3],[4]. The presence of even a single extra chromosome in primary mouse embryonic fibroblasts (MEFs) results in proliferative defects and metabolic aberrations [5]. In *Caenorhabditis elegans* and *Drosophila melanogaster*, aneuploidy is often lethal [6],[7], and in budding and fission yeasts, aneuploidy can inhibit cellular proliferation [8],[9].

However, aneuploidy can be advantageous in fungi by conferring antifungal drug resistance and enabling rapid adaptive evolution. Aneuploidy evokes transcriptomic and proteomic changes in the model yeast *Saccharomyces cerevisiae* [8],[10]. Mutations in a deubiquitinating enzyme of *S. cerevisiae*, which arose during the evolution of an aneuploid isolate, leads to improved proliferation of many aneuploid strains, likely by promoting degradation of aberrant proteins produced in perturbed stoichiometric ratios [11]. Rancanti et al. found that aneuploidy facilitated adaptive evolution in yeast cells lacking the conserved motor protein Myo1 involved in cytokinesis [12]. Moreover, it has been found that chromosomal duplication may confer a selective advantage to *S. cerevisiae* under stress conditions by promoting genomic instability and mutation, and although it is a transient solution, the short-lived aneuploid intermediate may also serve as a capacitator of evolution [13],[14]. In the human pathogenic fungi *Candida albicans* and *Cryptococcus neoformans*, aneuploidy can confer resistance to commonly used antifungal drugs such as fluconazole [15]–[18]. In *C. albicans*, haploids can even arise from concerted chromosome loss from diploid progenitors [19]. In *C. albicans*, an isochromosome 5 can arise in response to fluconazole treatment and confers drug resistance because the left arm of Chr 5 encodes Erg11 (lanosterol 14α demethylase), the target of fluconazole, and Tac1, a transcription factor that activates expression of drug export pumps [15],[17]. Similarly in *C. neoformans*, Chr 1 disomy confers azole resistance because this chromosome harbors *ERG11* and *AFR1* (which encodes the major azole efflux pump) and aneuploidy can also influence the virulence of this pathogen [16],[20],[21]. Interestingly, a similar drug-resistance phenotype has been observed in the protozoan parasite *Leishmania*, in which resistance to front line antimonial-based drugs similarly emerges via aneuploidy [22],[23].

*C. neoformans* is a globally distributed human fungal pathogen that causes life-threatening meningoencephalitis [24]. *Cryptococcus* predominantly infects individuals with compromised immunity, such as HIV/AIDS patients. The United States Centers for Disease Control (CDC) has reported that *C. neoformans* causes more than one million cases of cryptococcosis annually with more than 620,000 attributable mortalities, resulting in approximately one-third of all AIDS-associated deaths [25]. This fungal pathogen has now surpassed tuberculosis as a common cause of death in Africa.

*C. neoformans* is a basidiomycetous fungus that usually grows in the environment as a haploid, budding yeast. This species has a bipolar mating system with two mating types: **a** and α [26]–[28]. In response to a variety of environmental conditions, **a** and α cells secrete lipid-modified pheromones that induce cell–cell fusion, and the resulting dikaryon undergoes a dimorphic transition to hyphal growth [28]–[31]. Ultimately, the hyphal tips form basidia fruiting bodies wherein nuclear fusion and meiosis occur, and multiple rounds of mitosis and budding result in the production of four long chains of infectious spores decorating each basidium. These spores are readily aerosolized and cause infections in humans and animals when inhaled [32].

While *C. neoformans* has a defined **a**-α opposite sexual cycle, >99% of natural isolates are of the α mating type [33]. *C. neoformans* var. *neoformans* (serotype D) α cells can undergo α-α unisexual reproduction to generate spores under laboratory conditions [34]. Recent population genetic studies provide evidence that multiple pathogenic lineages of *C. neoformans* var. *grubii* (serotype A) and the sibling species responsible for the Vancouver outbreak *Cryptococcus gattii* (serotype B and C) undergo α-α sexual reproduction in nature, but this remains to be documented under laboratory culture conditions [30],[35]–[41]. Under nutrient-limiting conditions α haploid cells form a diploid or a haploid monokaryotic hyphae. The haploid hyphae grow to form basidia where a late diploidization event occurs. In the diploid hyphae diploidization of the nuclear content may be induced early and results in a diploid monokaryon that initiates hyphal growth and the formation of apical basidia. Early diploidization may occur through endoreplication or cell–cell fusion. Cell fusion, followed by nuclear fusion, may be induced in ménage à trois matings where **a** cells serve as pheromone donors to stimulate α-α fusion between genetically different or clonal cells [34]. Endoreplication may occur in cells that undergo DNA replication without cell division or they may undergo nuclear division and then fusion. In all cases, the resulting diploid nucleus undergoes meiosis and multiple rounds of mitosis generate the meiotic progeny, the basidiospores. In previous studies, we found that the key meiotic regulators, the endonuclease Spo11 and the meiotic recombinase Dmc1, are required for spore production and germination during unisexual reproduction [34],[42]. Deletion of *SPO11* or *DMC1* severely impairs sporulation and the fewer meiotic progeny generated are largely inviable, providing further evidence that unisexual reproduction is a meiotic process [34],[42].

Remarkably, unisexual reproduction has been recently discovered to occur in another common systemic human fungal pathogen, *C. albicans* [43]. *C. albicans* is known to undergo a parasexual cycle involving heterothallic fusion of α and **a** mating type cells followed by stochastic, concerted loss of chromosomes [44]. Similarly, fusion of α-α and **a**-**a** cell unions via homothallic parasexual reproduction occurs when a pheromone-degrading protease (Bar1) is inactivated or in ménage à trois matings, in which a third mating partner serves only as the pheromone donor [43].

Sex is costly, and thus the question arises as to why *C. neoformans* or *C. albicans* would undergo inbreeding/selfing unisexual reproduction, which would limit the amount of genetic diversity inherited from the parents, as opposed to **a**-α sexual reproduction, which promotes outcrossing and genetic exchange. We hypothesize that unisexual reproduction is a hypermutagenic process that generates genetic diversity *de novo* and that the resulting progeny can thereby more rapidly adapt to changing environments than cells produced asexually by mitosis.

To test this hypothesis, we isolated progeny generated via selfing α-α unisexual reproduction and subjected them to phenotypic and genotypic analyses. Remarkably, we found that α-α unisexual reproduction generates frequent phenotypic diversity, including temperature sensitivity, fluconazole resistance or sensitivity, and increased melanin production among other novel traits. Further comparative genomic hybridization (CGH) analysis revealed that the majority of phenotypically diverse progeny are aneuploid. Phenotypic diversity is caused by this aneuploidy, as loss of the aneuploid chromosomes restored both euploidy and the wild-type parental phenotype. Aneuploidy and phenotypic variation was also found to occur at a similar rate following **a**-α bisexual reproduction but not as a result of mitotic asexual vegetative growth. Our findings show that sex can generate phenotypic and genotypic diversity *de novo* in the pathogenic yeast *C. neoformans* with implications for other eukaryotic microbes and pathogens, including other fungi and parasites that are common pathogens of humans.

## Results

### Genomic Comparison of Parental Strain JEC21 and Its Hyperunisexual Selfing Progeny XL280

To investigate whether phenotypic and genotypic changes are generated by selfing unisexual reproduction, we analyzed the meiotic progeny produced via this process. To this end, we solo cultured the promiscuous, hypersexual haploid strain XL280α under conditions that support robust α-α unisexual reproduction (V8 media or FA (filament agar) for ∼2 weeks) and isolated spores by microdissection. The hyperfilamentous haploid strain XL280α [34],[45] generates abundant hyphae, homozygous diploid intermediates, and haploid meiotic spores when grown on mating-inducing media all by itself.

**Figure 1:**
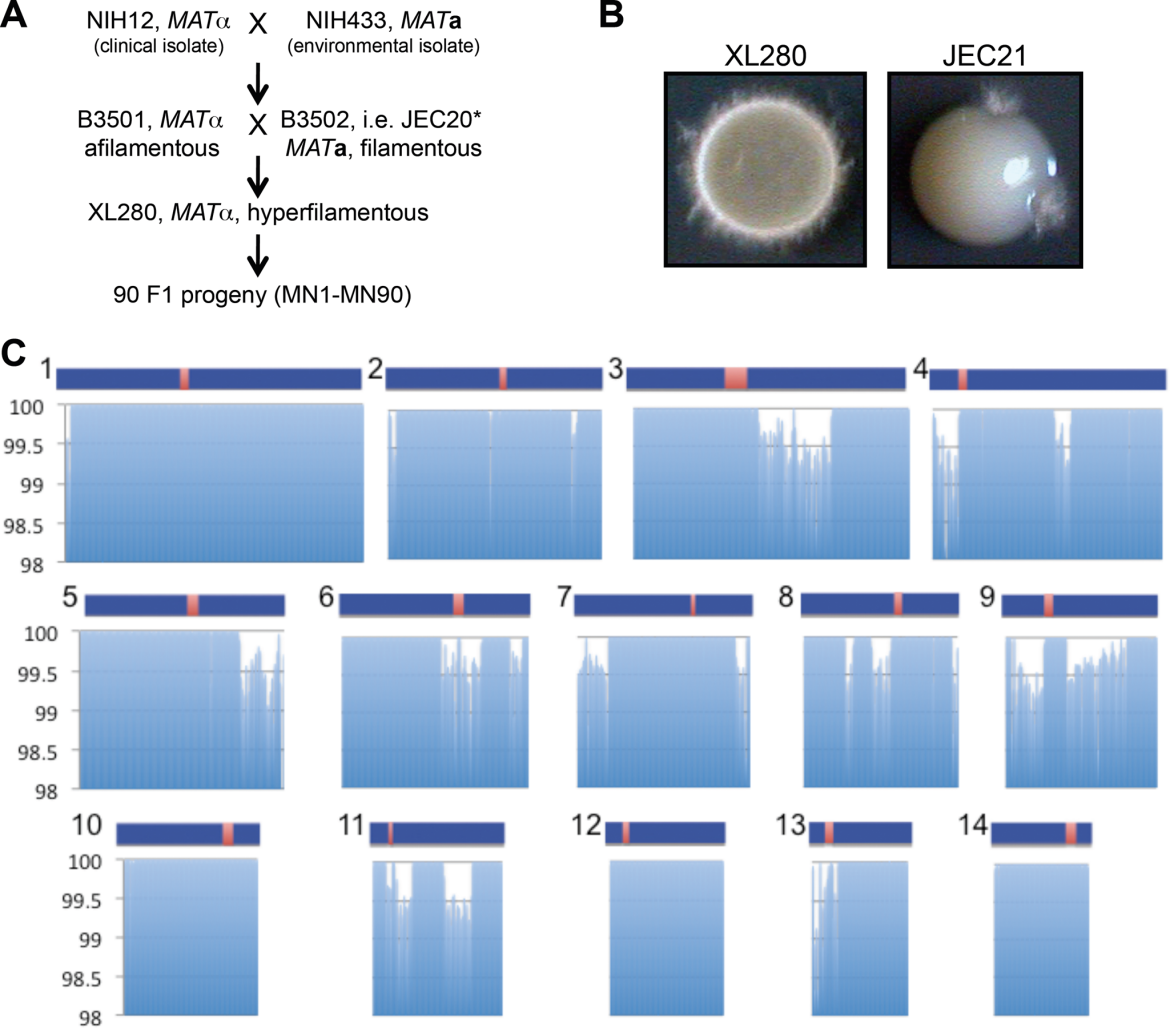
Phenotypic and genomic comparison between XL280 and JEC21. (A) The origin of XL280. The parents are NIH12 (*MAT*α), a clinical isolate from the United States, and NIH433 (*MAT* **a**), an environmental isolate from Denmark. * indicates that genome sequences are available. (B) XL280 is hyperfilamentous. α-α unisexual reproduction generates hyphae, basidia, and meiotic spores along the periphery of yeast colonies incubated on filament agar (FA) at room temperature for 11 d. (C) Whole genome comparison between XL280 and JEC21. Distribution of sequence identity (*y*-axis) across the genomes averaged over 10 kb intervals. Blue bars represent the 14 chromosomes of JEC21 [46], and red regions represent the candidate centromeres [69].

Xl280 is a haploid F1 progeny descended from a cross between two exceptionally well-validated haploid sibling parental strains, whose genomes have been both sequenced, B3501α and JEC20**a** (which is isogenic to the sequenced strain JEC21α, with the exception of the mating-type locus) (Figure 1A) [34],[45]–[47]. Thus, while XL280α shares markers with both parents, it only has one copy of each chromosome (it is haploid) and is not heterozygous anywhere in its genome. When it undergoes unisexual reproduction with itself, it forms a transient homozygous diploid (generated through either α-α cell fusion between mother and daughter cell or via endoreplication) that is identical throughout the diploid, euploid genome, in which each gene is present in two identical copies.

Hyphae produced by unisexual reproduction grow to form terminal basidia wherein the diploid nucleus undergoes meiosis to generate abundant recombinant progeny. That this is a meiotic process has been established in previous studies [34],[42] that showed both key meiotic genes *SPO11* and *DMC1* are required for spore production and viability. The parental strains B3501α and JEC20**a** share ∼50% genetic identity [46]; thus, their F1 progeny (XL280) would be expected to share ∼75% genetic identity with both B3501α and JEC21α, whose genomes have been sequenced [46]. Therefore, we used the available JEC21 genomic sequence and genome microarray slides as a foundation to study the genome of XL280 and its α-α unisexual reproduction [48],[49].

We performed next-generation sequencing (NGS) using high-throughput Illumina sequencing to compare the genomes of XL280α and JEC21α [50]. In total, 47,891,302 sequences (paired-end reads) of 75 bp in length were generated, providing 160-fold coverage of the XL280 genomic sequence, which was then analyzed using the known JEC21 genome as the reference for the sequence assembly (Figure S1 and Table S1). All but 4,535,426 sequence reads (∼10%) were used in the genome assembly, and of these remaining reads, the majority were of low quality (90%). We assembled the Illumina reads using a combination of *de novo* assembly (Velvet, [51]) and reference genome assembly (BWA, [52]) (see Figure S1 and Materials and Methods for detailed procedure).

XL280 shares 100% identity with JEC21 over 81% of the overall genome and is 99.88% identical at the sequence level overall (Figure 1C). Among the SNPs and small indels identified, 9,021 were located in exonic regions, 10,254 in intergenic regions, nine in tRNA genes, and 3,849 in intronic regions (31 of which differed in the 5′ splice-site and 23 in the 3′ splice-site) (Table S2). Among the exonic SNPs and indels, 107 SNPs resulted in the introduction or deletion of stop codons, 68 indels resulted in a frame shift, 14 indels resulted in the loss of amino acids without a frame shift, and 3,943 SNPs resulted in nonsynonymous amino acid substitutions in 960 ORFs (Table S2). Further analysis of the modified ORFs will enable the elucidation of the phenotypic differences between XL280 and JEC21, for example with respect to the hypersexual phenotype [45].

**Figure 2:**
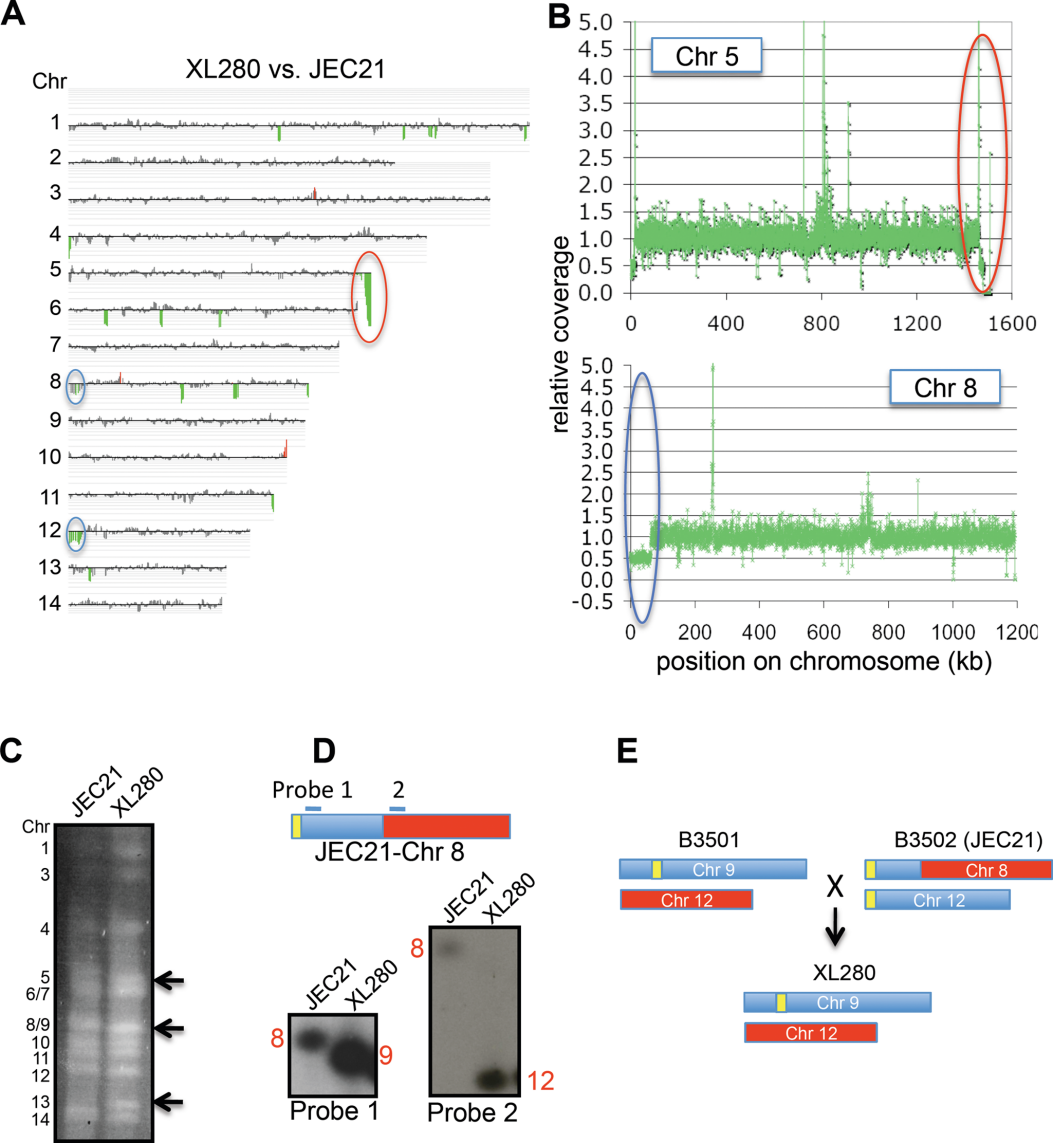
Genome comparison of XL280 and JEC21. (A) CGH of XL280 versus JEC21. Coloring indicates gene dosage as follows: gray, no significant change; red, more abundant; green, less abundant. (B) Coverage analysis of XL280 Chr 5 and Chr 8 with JEC21 as the reference. The red circle indicates that XL280 lacks ∼28 kb near the right end of Chr 5 compared to JEC21. The blue circle indicates that XL280 has one copy of the 63 kb region that is duplicated in JEC21 and located on the left ends of Chr 8 and Chr 12. (C) Chromosome configuration of XL280 versus JEC21. Black arrows indicate the size differences of Chr 5, 8/9, and 13 between the two strains. (D) Two probes located before and after the break in Chr 8 of JEC21 hybridized to XL280 Chr 9 and 12, respectively. (E) Chr 9 and 12 of XL280 versus JEC21.

CGH was performed using a 70-mer oligonucleotide microarray covering all predicted ORFs of the JEC21 genome [49] to detect chromosome copy number alterations. Based on CGH, the genomes of XL280 and JEC21 (Figure 2A) are quite similar and span 14 conserved, linear chromosomes. This enabled use of JEC21-based microarray slides for CGH analysis of XL280 unisexual progeny. Because this microarray covers only ORFs, no differences could be detected in centromeric or telomeric regions. There is an ∼28 kb region absent in XL280; in JEC21 this sequence lies near the right end of Chr 5 and is conserved in many *C. neoformans* strains [53]. In addition, XL280 is missing one copy of the left end of either Chr 8 or 12, which is a 63-kb duplicated region located on both Chr 8 and 12 of JEC21 [54]. NGS data further confirmed these findings (Figure 2B).

The chromosome content of strain XL280 was compared to JEC21 by clamped homogenous electric field (CHEF) gel electrophoresis to detect any chromosomal translocations. Chr 5 and Chr 8/9 of XL280 are smaller than those of JEC21, while Chr 13 of XL280 is larger (Figure 2C). To ascertain where the sequence similarity of these chromosomes lies, we excised each chromosomal band and performed band array analysis. The configurations of Chr 9 and 12 differed between XL280 and JEC21 (Figure S2). Chr 9 of XL280 hybridized to Chr 8 and Chr 12 of JEC21; Chr 12 of XL280 hybridized to Chr 8 of JEC21. These findings were confirmed by Southern hybridization (Figure 2D). Previous studies reported that this chromosomal translocation and duplication occurred during generation of the JEC21α/JEC20**a** congenic strain pair [54]. During the cross of strains B3501α×B3502**a**, Chr 9 and Chr 12 formed a dicentric chromosome via telomere–telomere fusion and then broke to form new versions of Chr 12 and Chr 8 in strains JEC21/JEC20 [54]. Our results indicate that Chr 9 and Chr 12 of XL280 are more similar to those of the B3501α parent, whereas other chromosomes are more similar to the JEC20**a** parent (Figure 2E). This comprehensive analysis of the XL280 genome reveals how its genome is derived from two well-validated parental reference strains and allowed detailed molecular analyses of unisexual progeny.

### Frequent Phenotypic and Genotypic Changes Following *C. neoformans* Unisexual Reproduction

**Figure 3:**
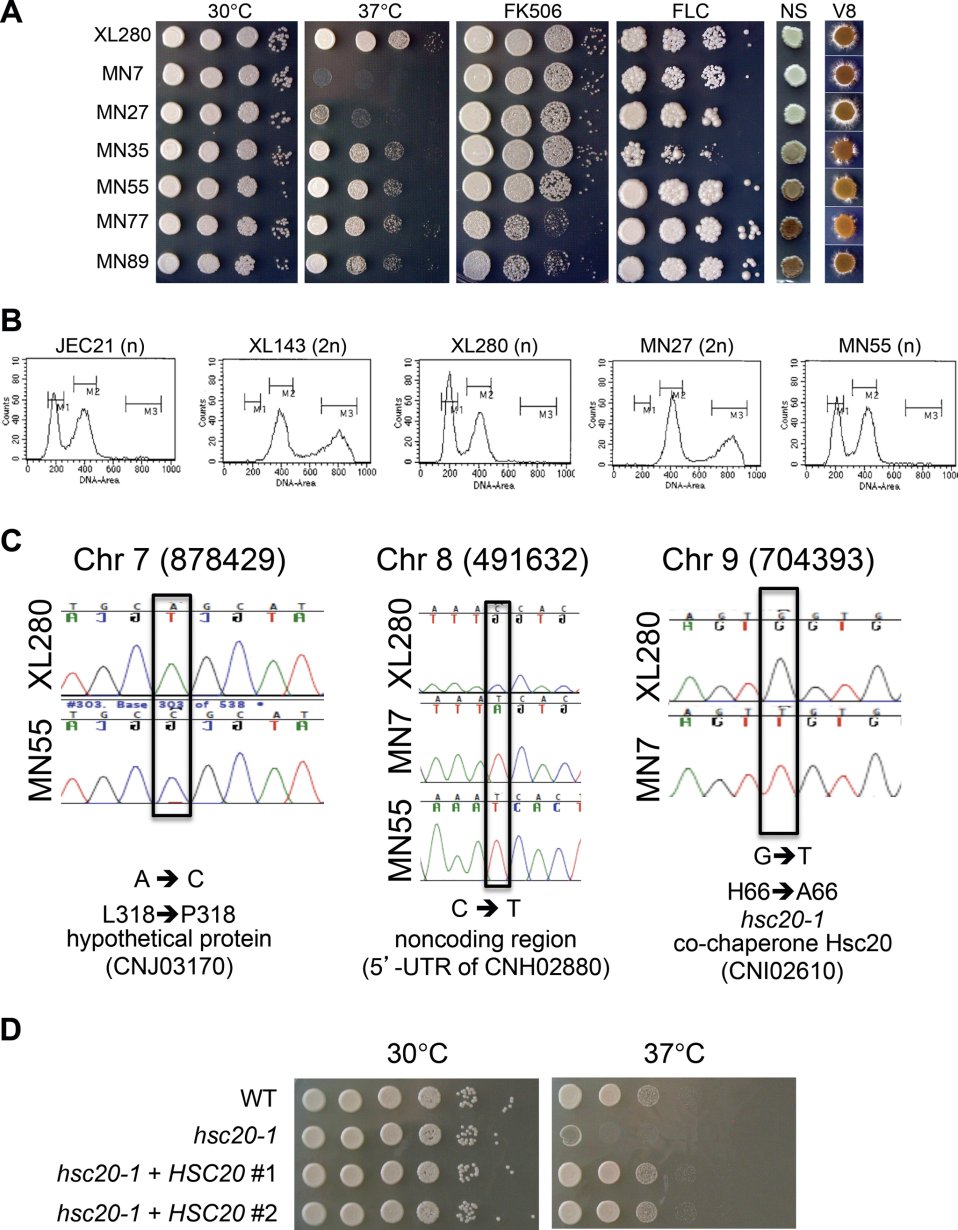
Unisexual reproduction generates phenotypic diversity. (A) Progeny produced by α-α unisexual reproduction of strain XL280 in solo cultures were grown in 10-fold serial dilution assays and under the following conditions: YPD at 30°C for 2 d; YPD at 37°C for 2 d; YPD plus 1 µg/mL FK506 at 30°C for 2 d; YPD plus 8 µg/mL fluconazole (FLC) at 30°C for 6 d; NS (melanin-inducing media) at 30°C for 3 d; and V8 pH = 7 (mating-inducing media) at room temperature for 11 d. (B) Diploid progeny were generated infrequently following unisexual reproduction. Flow cytometry profiles of cells stained with the fluorescent dye propidium iodide. JEC21 (1n, haploid control); XL143 (2n, diploid control); XL280 (1n); MN27 (2n, the only diploid strain identified among the 90 XL280 α-α unisexual reproduction progeny (1.1%). All other XL280 α-α unisexual progeny were haploid (or aneuploid) (e.g., MN55). Nuclear DNA content is indicated by 1n (haploid) and 2n (diploid). The *x*-axis indicates fluorescence intensity reflecting DNA content, and the *y*-axis indicates cell counts. (C) Three SNPs identified by NGS from strains MN7 and MN55 and were confirmed by Sanger sequencing. (D) The WT allele of *HSC20* was cloned in two independent plasmids, and these were used to transform strain MN7 and independent transformants were analyzed. This complementation test shows that the TS phenotype of MN7 is attributable to the recessive *hsc20-1* mutation.

Solo culture of strain XL280 on V8 sexual reproduction-inducing media resulted in robust production of F1 meiotic spore progeny via selfing α-α unisexual reproduction. This process involves ploidy changes (1n→2n→1n) via self cell–cell fusion or endoreplication, meiosis, and sporulation to produce haploid meiotic progeny. In total, 90 α-α unisexual reproduction meiotic progeny were isolated by spore micromanipulation and germination and were examined in a battery of phenotypic analyses. We assessed major virulence factors of *C. neoformans* (growth at 37°C and melanin production on niger seed (NS) or L-DOPA media), sensitivity to the antifungal drugs fluconazole (FLC) or FK506, and unisexual reproduction (self-filamentous growth) (Figure S3). In these analyses, six of 90 F1 progeny (∼7%) showed a distinct phenotype compared to the haploid parental strain XL280 and the remaining 84 progeny showed no phenotypic differences from the wild-type (Figure 3A and Figure S3). The six isolated variant F1 progeny strains all exhibited temperature-sensitive (TS) growth (Figure 3A). Two progeny (MN77 and MN89) were sensitive to the calcineurin inhibitor FK506 and produced more melanin, indicating they may have similar genetic changes. MN35 was FLC-sensitive, while the other four progeny (MN27, MN55, MN77, and MN89) were relatively FLC-resistant (Figure 3A). On V8 mating-inducing media (V8 agar), MN27 underwent more robust unisexual development, while MN35, MN55, MN77, and MN89 generated fewer hyphae (Figure 3A). Although the two genomes of cells undergoing α-α unisexual reproduction are identical, phenotypic changes to these and other conditions (Figure S4) are clearly evident in even a relatively small sample of progeny produced by this process, which indicates that α-α unisexual reproduction generates phenotypic plasticity *de novo* since there is no preexisting genetic diversity to admix.

We next tested if standard mitotic growth might also lead to phenotypic changes like meiotic unisexual reproduction. A similar set of 96 isolates derived from XL280 by mitotic asexual growth exhibited no phenotypic variation from wild-type in the same battery of phenotypic tests (Figure S5) in which phenotypic variation was readily detected in meiotic unisexual reproduction. Thus, we conclude that phenotypic variation occurs following meiotic unisex but not standard mitotic asexual growth.

Phenotypic variation is often attributable to genetic changes. To establish the molecular basis of phenotypic variation of the unisexual progeny, we examined changes in ploidy, whole-chromosome aneuploidy, chromosomal translocations, and single nucleotide polymorphisms (SNPs) by FACS, CGH, CHEF, and Illumina NGS, respectively. FACS analysis revealed that the hypersexual progeny MN27 was diploid, in accord with previous findings that diploid strains are (1) intermediates or products of unisexual reproduction and (2) hyperfilamentous (Figure 3B) [34]. None of the other 90 F1 α-α unisexually produced progeny were diploid (Figure 3B and unpublished data).

Three progeny (MN7, MN55, and MN89) were analyzed by Illumina NGS to examine the presence of SNPs. In total, only three SNPs were identified in the ∼20 Mb genomes of the three isolates based on 48,644,802 sequences of 75 bp generated for MN7, 46,089,962 for MN55, and 44,650,780 for MN89 (Figure 3C). One SNP located at position 878,429 on Chr 7 of MN55 resulted in an L318P coding substitution in the hypothetical protein CNJ03170. A second SNP was located at position 491,632 on Chr 8 in both MN7 and MN55, resulting in a cytosine to thymine change in the 5′ UTR of the CNH02880 gene. The third SNP was located at position 704,393 on Chr 9 of MN7 and resulted in an H66A amino-acid substitution in the co-chaperone Hsc20 (CNI02610), a heat shock protein that functions in iron–sulfur cluster assembly [55]. To examine whether this SNP is responsible for the TS phenotype of isolate MN7, we performed a complementation test by introducing the WT allele of *HSC20* into the MN7 TS strain. We first documented that the *hsc20-1* mutant allele is recessive based on analysis of an *hsc20-1*/*HSC20* diploid fusion product of MN7 and XL280α. While the MN7 strain is TS, the MN7×XL280α diploid fusion product was temperature resistant, similar to the XL280α parent. The WT allele of *HSC20* was cloned in a plasmid under control of its native promoter and terminator and introduced into strain MN7 by biolistic transformation. In multiple independent transformants, we found that a single copy of the WT *HSC20* allele complemented the TS phenotype of MN7 and restored WT growth similar to the XL280α parent at higher temperature (Figure 3D). Based on these findings we conclude that the TS phenotype of progeny MN7 is attributable to the H66A amino-acid substitution in the co-chaperone Hsc20.

**Figure 4:**
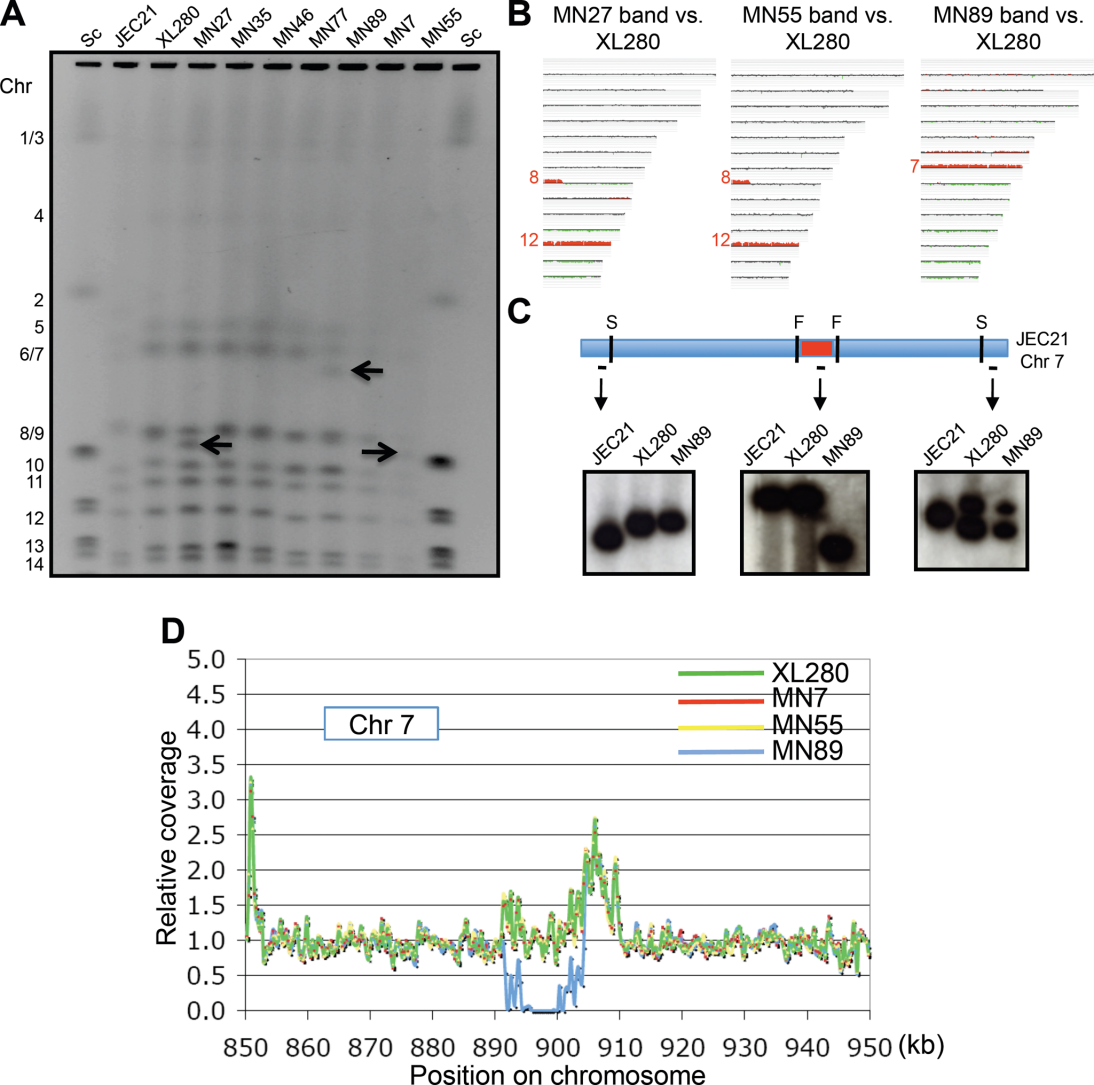
Chromosome deletions arise during unisexual reproduction. (A) PFGE molecular karyotyping of parental strain XL280 and 7 α-α unisexual produced progeny. Chromosome numbers are indicated on the left. Arrows emphasize the chromosomes that differ in size in progeny strains MN27, MN55, and MN89 compared to parental strain XL280. Sc represents the 0.225 to 2.2 Mb *S. cerevisiae* CHEF DNA Size Markers (BioRad 170-3605). (B) Band array data for chromosomes indicated with black arrows in panel A. DNA of the labeled chromosome band was extracted from the PFGE gel and subjected to CGH. Coloring indicates gene dosage as follows: gray, no significant change; red, more abundant; green, less abundant. (C) A shorter centromere is observed in progeny strain MN89 based on Southern blot analysis using restriction enzymes SwaI and HpaI. (D) The shorter centromere of MN89 was confirmed by genome sequencing. An ∼10,257-bp region (blue line) is missing in progeny MN89 compared with parent XL280.

Based on CHEF analysis of the chromosomal karyotype, chromosome size differences were detected in the α-α unisexual F1 progeny MN27, MN55, and MN89 (Figure 4A). For MN27 and MN55, an extra chromosomal band was present and migrated more rapidly than Chr 8/9. For MN89, an extra chromosomal band was observed to migrate more rapidly than Chr 6/7. To further analyze these anomalous chromosomes, we excised the novel chromosomal bands from the CHEF gel and extracted and fluorescently labeled the DNA to generate probes. Band CGH analysis revealed that the excised chromosomal bands covered all ORF regions (the JEC21 expression array covers ORF and not centromeric or telomeric regions) of Chr 9, Chr 9, or Chr 7 in isolates MN27, MN55, or MN89, respectively (Figure 4B) (XL280 Chr 9 hybridizes to Chr 8 and 12 on the JEC21 array). Because all ORFs were recognized despite a shortened chromosomal length, we hypothesized that centromeric or telomeric regions (not represented on the array) may have suffered significant deletions in the respective strains. To test this hypothesis, we chose MN89 as an example and designed specific probes that hybridize to the centromeric and telomeric regions of Chr 7 (Figure 4C). Restriction enzyme digestion of whole chromosomes and CHEF electrophoresis followed by Southern hybridization revealed that Chr 7 of MN89 has the same telomere lengths as those of XL280, but a shortened centromeric region (Figure 4C). Further whole genome sequencing determined that the size of the deleted centromeric region is 10,257 bp (Figure 4D).

These findings indicate that α-α unisexual reproduction induces phenotypic and genotypic plasticity. However, a common genetic change responsible for the phenotypic changes remained to be identified.

### α-α Unisexual Reproduction Frequently Generates Aneuploidy

**Figure 5:**
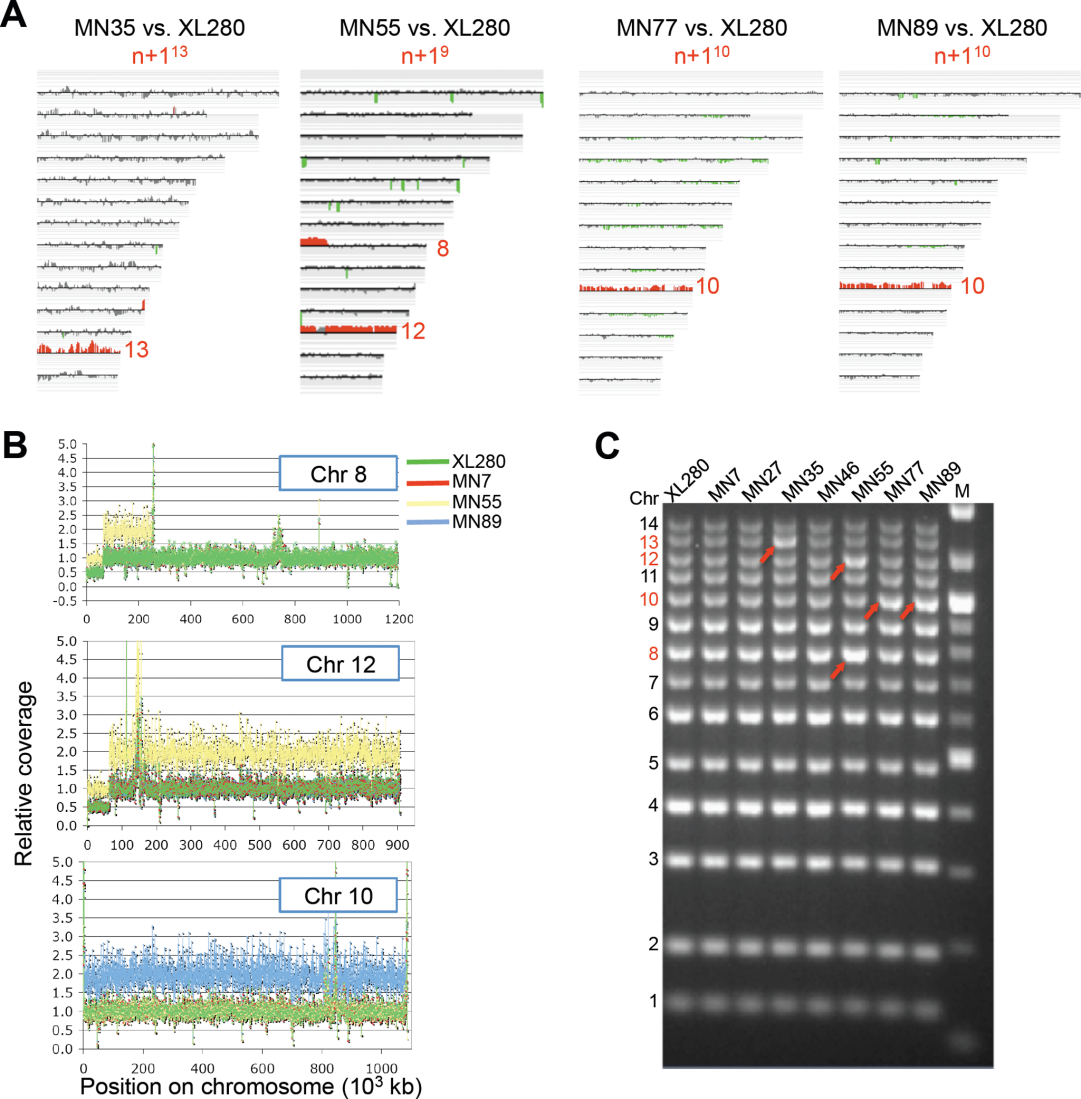
Aneuploidy is generated at a high rate by unisexual reproduction. (A) CGH array data for four progeny with novel phenotypes. Progeny strains MN35, MN55, MN77, and MN89 contain an extra copy of Chr 13, Chr 9, Chr 10, and Chr 10, respectively. The colors indicate gene dosage as follows: gray, no significant change; red, more abundant; green, less abundant. (B) NGS is consistent with the CGH data in that 2× coverage was observed for each aneuploid chromosome and no others. MN55 (yellow) has an extra copy of Chr 9, which corresponds to the left arm of Chr 8 and the right arm of Chr 12 of strain JEC21 (as shown above). MN89 (blue) has an extra copy of Chr 10. (C) Multiplex PCR was conducted to detect aneuploidy. Multiplex PCR reactions used the primer sets listed in Table S3. Amplicons (i.e., chromosomes or chromosome regions) that differed in abundance between samples were identified in this assay and quantified by scanning (arrowheads). Chromosome numbers are indicated on the left.

CGH analysis of the phenotypically variant progeny revealed that four out of six isolates (66.7%) were aneuploid. Isolates MN35, MN55, MN77, and MN89 contained an extra copy of chromosome 13, 9, 10, and 10, respectively, indicating that a high rate (>4%) of aneuploidy is generated as a consequence of α-α unisexual reproduction (Figure 5A). Whole genome sequencing of isolates MN55 and MN89 confirmed the presence of an extra copy of Chr 9 or Chr 10, respectively, in a majority of cells in the population, as ∼2-fold higher levels of sequence reads were obtained for these chromosomes compared to other genomic regions (Figure 5B). Interestingly, Sionov et al. previously associated Chr 10 disomy with FLC resistance [16].

As a complementary approach to CGH, we developed a facile multiplex PCR-based method to detect aneuploidy. In this approach, 14 primer pairs (Table S3) were combined into a single PCR reaction such that each primer pair represents a separate chromosome and amplifies a specific region of different sizes to generate a ladder of 100 to 1400 bp (Figure 5C). PCR products of greater intensity reflect the presence of extra chromosomes (red arrows in Figure 5C). Bioanalyzer analysis further confirmed a ∼2-fold increase in PCR product yield from the additional aneuploid chromosomes present in 1n+1 isolates (Figure S6). Using multiplex PCR we screened all of the XL280 meiotic (90) and mitotic (96) progeny and we did not detect any additional aneuploid strains from the meiotic progeny and none from the mitotic progeny (Figure S7 and Figure S8).

In *S. cerevisiae*, strains aneuploid for different chromosomes all share several common phenotypes, including temperature sensitivity [8]. Therefore, we determined whether aneuploid strains of *Cryptococcus* exhibit a similar phenotype. Consistent with the observed consequences of aneuploidy in *S. cerevisiae*, all aneuploid *C. neoformans* strains in our study exhibited a TS phenotype (Figure 3A).

**Figure 6:**
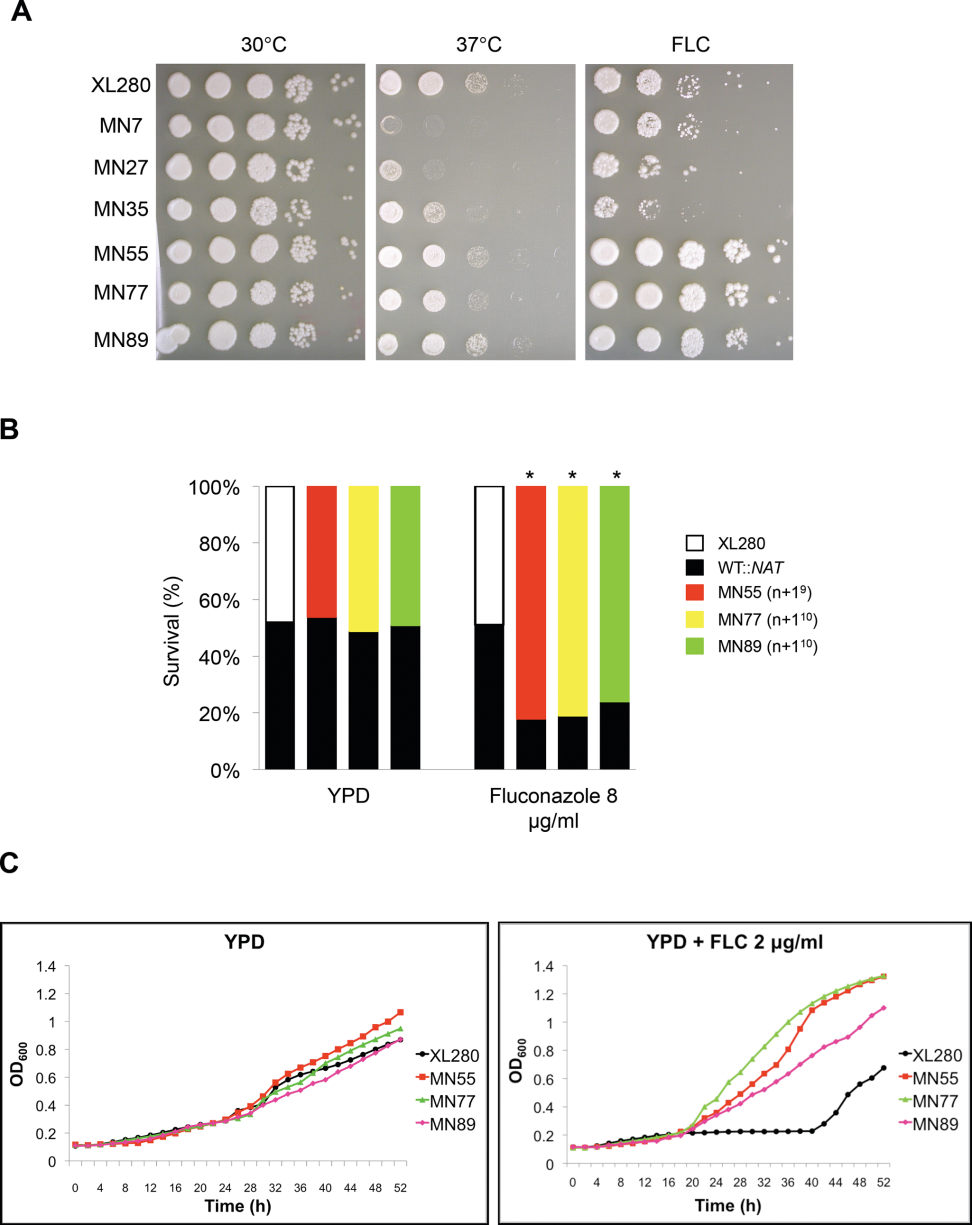
Aneuploid strains are fluconazole resistance. (A) XL280 meiotic progeny were grown in YPD, diluted 10-fold in water, spotted, and incubated under the following conditions: YPD at 30°C for 2 d, YPD at 37°C for 2 d, and YPD plus 8 µg/mL fluconazole (FLC) at 30°C for 6 d. (B) Three aneuploid strains exhibited increased competitive fitness in competition assays. An XL280 strain marked with a *NAT* resistance marker integrated in the *SPO11* genetic locus was mixed together at equal abundance with XL280, MN55 (n+1^9^), MN77 (n+1^10^), or MN89 (n+1^10^). The mixtures were incubated on YPD and YPD plus 8 µg/mL fluconazole (FLC) at 30°C for 3 d. The cells were scraped from the plates, serially diluted, and plated on YPD and YPD plus NAT. The ratios of survival of each strain are shown for one representative experiment, and * represents significance at *p*<0.005 from three independent experiments. We note that the *NAT* marker was neutral in this analysis and served to mark and detect the WT competitor cells. (C) The three aneuploid strains exhibited higher growth rates in the presence of fluconazole. Growth curves of the aneuploid strains were similar to the wild-type in rich media. However, in the presence of fluconazole the growth rate of the aneuploid strains was significantly higher than the wild-type.

Aneuploidy can compromise growth unless it provides a fitness benefit under stress or other distinct conditions. We found that aneuploid strains exhibit a variety of phenotypes potentially associated with virulence, including antifungal drug resistance. To test if the progeny have a competitive advantage relative to the parental strain, we co-incubated an aneuploid progeny (MN55, MN77, or MN89) with the wild-type parent XL280α (marked with the NAT drug resistance marker) in the presence of fluconazole. As expected, the fluconazole-resistant aneuploid progeny exhibited an increased competitive fitness and outgrew the parental strain in competitive growth assays by a ratio of ∼4∶1 (Figure 6).

**Figure 7:**
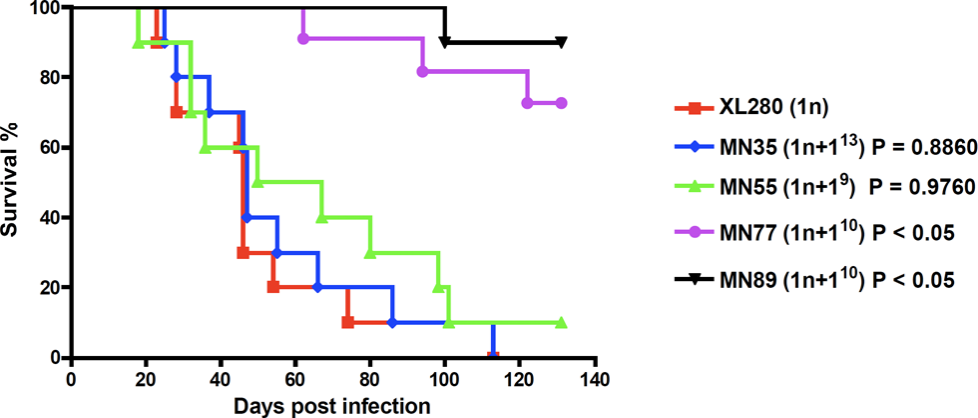
Aneuploid strains are pathogenic in the murine inhalation model. Ten female DBA mice per group were infected with 10^6^ cells of aneuploid strains MN35, MN55, MN77, and MN89. The mice were anesthetized by intraperitoneal injection of phenobarbital, and they were infected through intranasal instillation. The animals were monitored daily for clinical sighs of cryptococcal infection and sacrificed at predetermined clinical points that predict imminent mortality.

Moreover, to investigate the fitness of these aneuploid unisexual progeny, we tested their ability to infect and cause disease in a murine inhalation model. Mice were infected with the aneuploid strains via intranasal instillation and monitored for signs of pulmonary cryptococcosis or meningoencephalitis. In contrast to the view that aneuploidy is deleterious, the aneuploid unisexually generated isolates MN35 (n+1^13^) and MN55 (n+1^9^) were as virulent as the euploid wild-type parent, despite their modest TS growth at 37°C on YPD rich media *in vitro* (Figure 7). Previous studies have shown that in the clinically important sibling species *C. neoformans* var. *grubii* (serotype A) strains that are disomic for chromosome 13 exhibit a melanin defect and impaired virulence in mice [21]. However, we found that disomy 13 in *C. neoformans* var. *neoformans* (serotype D) did not affect melanin production and the MN35 strain was equally virulent as the wild-type. Thus, the specific consequences of aneuploidy may differ between even closely related lineages. Two other aneuploid strains (MN77 and MN89) were both attenuated compared to wild-type and, interestingly, both are aneuploid for chromosome 10 (Figure 7). Cells isolated from lungs of infected animals exhibited relatively stable aneuploid phenotypes and were found to retain their aneuploid chromosome in 70–80% of isolates based on multiplex PCR [20 isolates were analyzed from each strain, 15 were aneuploid for MN35 (75%), 16 for MN55 (80%), 14 for MN77 (70%), and 15 for MN89 (75%) (Figure S9)]. Thus, under the stressful gauntlet of the host, several aneuploid progeny appeared as fit as the euploid parent whereas others were attenuated.

### Aneuploidy Is Responsible for Phenotypic Changes

**Figure 8:**
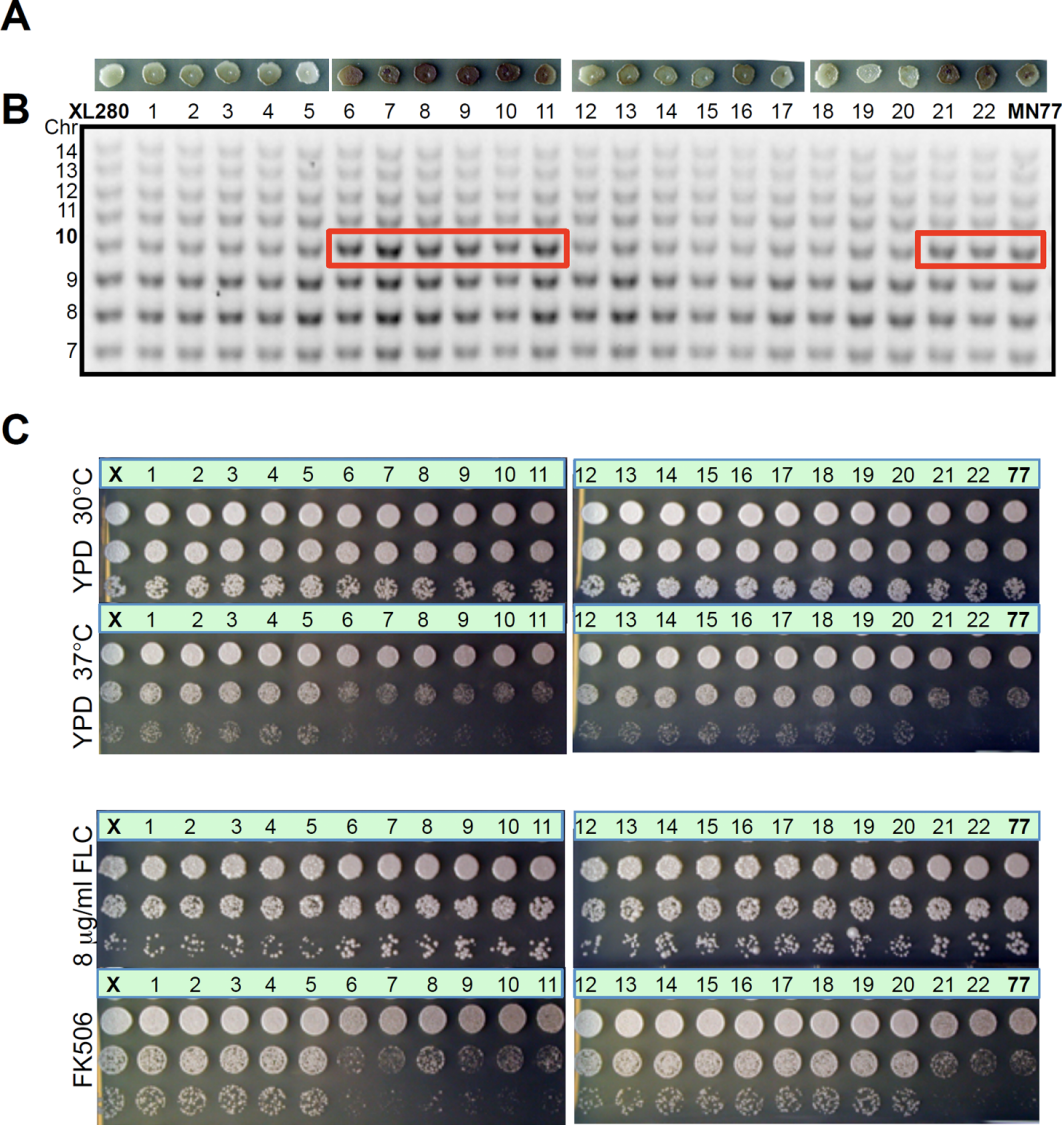
Aneuploidy causes the observed phenotypic changes. Strain MN77, which contains an extra copy of Chr 10 and has increased melanin production, was grown on YPD at 37°C for 3 d to promote aneuploidy loss. The genotypes and phenotypes of 22 resulting mitotic progeny were analyzed. (A) Melanin production of 22 mitotic progeny on NS media at 30°C for 2 d. (B) Multiplex PCR of 22 progeny detected an extra Chr 10 in all of the strains with the increased melanin phenotype and loss of the extra copy of Chr 10 in all progeny with a wild-type phenotype. (C) Phenotypic analysis of 22 mitotic progeny at high-temperature growth (37°C for 2 d) and with drug treatment (FLC and FK506). XL280 (X) and MN77 (77) are the WT and aneuploid controls, respectively.

To examine whether the observed phenotypic changes are a consequence of aneuploidy, we compared the phenotype of aneuploid strains and euploid derivatives obtained following loss of the extra chromosome. Aneuploid strains are relatively unstable and frequently lose the 1n+1 extra chromosomal copy to return to the 1n haploid euploid state. Here, we analyzed isolate MN77, which contains an extra copy of Chr 10 and produces increased amounts of melanin, a readily scorable phenotype. We grew MN77 at 37°C to promote loss of the extra chromosome and isolated 22 randomly selected colonies for phenotypic and genotypic testing (Figure 8). Of these 22 isolates, 14 lost the extra copy of Chr 10 based on multiplex PCR analysis (Figure 8B), and in all cases concomitantly lost the enhanced melanin production phenotype (Figure 8A), therefore suggesting that Chr 10 disomy is responsible for the higher level of melanin produced. The remaining eight isolates that retained the Chr 10 aneuploidy all continued to produce higher levels of melanin (Figure 8A). To test whether Chr 10 disomy is also correlated with other phenotypes, we performed growth dilution assays with fluconazole (at 30°C), FK506 (at 30°C), and for growth at 37°C. Similar to the melanin phenotype, all isolates that had lost the extra copy of Chr 10 exhibited WT phenotypes similar to the XL280 parent, and all isolates that retained the extra copy of Chr 10 exhibited the variant phenotype (Figure 8C). Similar experiments were performed for isolates MN35 (n+1^13^) and MN55 (n+1^9^), and we found that loss of the extra chromosome was linked with loss of the variant phenotypes (fluconazole sensitivity and fluconazole resistance, respectively) (Figure S10). These results indicate that aneuploidy causes the phenotypic changes observed in disomic strains.

### Aneuploidy Is Generated During Sexual Reproduction in *C. grubii* and *C. neoformans*

To test whether the generation of aneuploidy is specific to α-α unisexual reproduction, we isolated progeny from asexual mitotic reproduction and **a**-α opposite-sexual reproduction. We isolated 96 single colonies from yeast extract-peptone-dextrose (YPD) media following asexual mitotic reproduction and from spores produced on V8 agar following **a**-α sexual reproduction. No phenotypic or genotypic changes were detected among asexual mitotic progeny (0/96) (Figure S5 and Figure S8).

**Figure 9:**
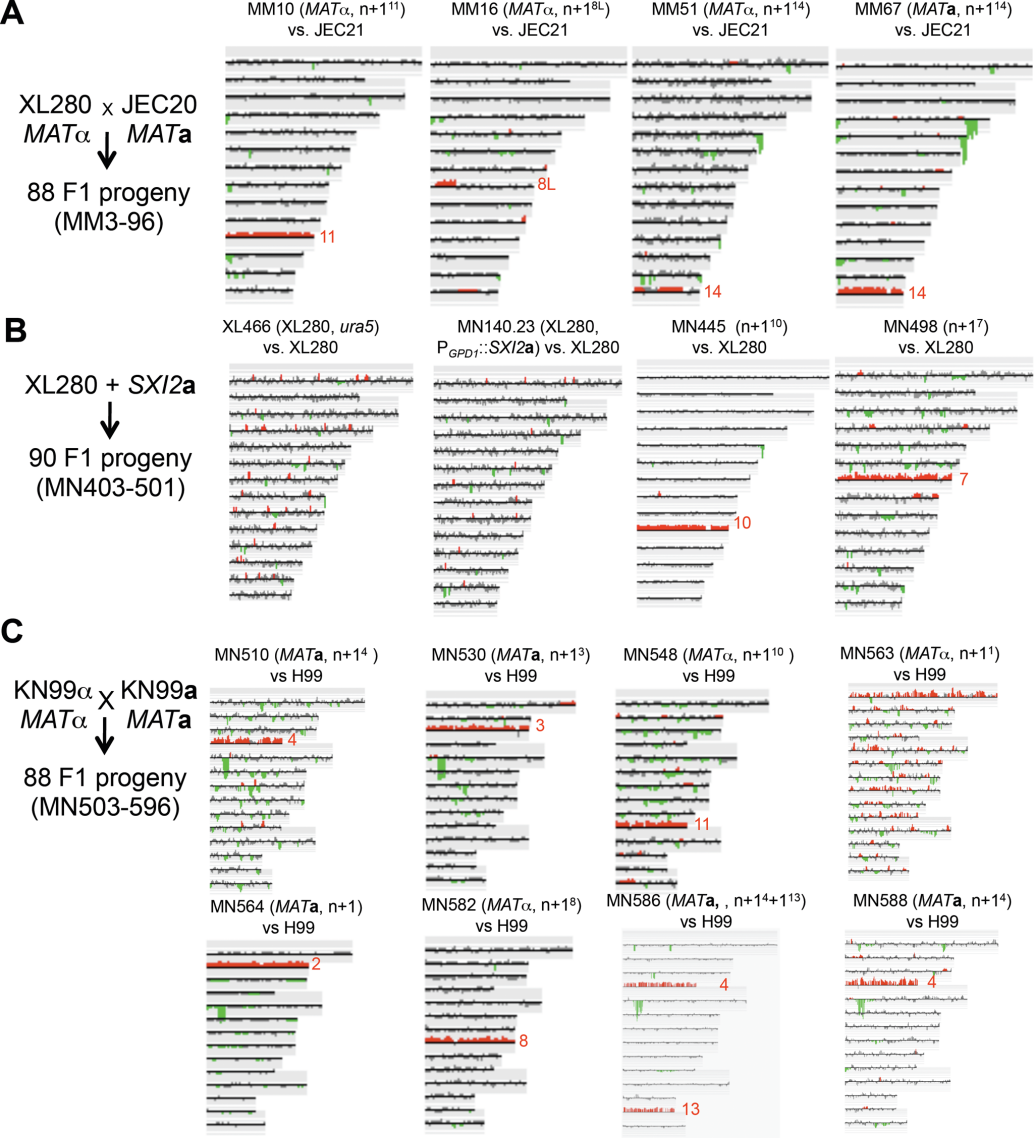
Aneuploidy is generated during a-α sexual reproduction. Genotypic analysis of representative progeny from (A) XL280α crossed with JEC20**a**; (B) XL280α “**a**-α” self-sexual reproduction generated via expression of *SXI2* **a**; and (C) *C. neoformans* var. *grubii* strain KN99α crossed with congenic strain KN99**a** [70].

To test **a**-α sexual reproduction, we first investigated 88 F1 progeny from the cross between strains XL280α and JEC20**a**. To reduce the chance that α-α unisexual progeny from XL280 were mixed with the **a**-α sexual reproduction progeny from the cross, three times more yeast cells from the **a** parent JEC20 were mixed with the XL280α cells in the cross. As these strains are ∼81% congenic, but not isogenic, they exhibit different phenotypes under various conditions, and their progeny (88 were examined) showed a range of phenotypes (Figure S11). Among 11 strains that had the most overt phenotypic changes, four were aneuploid and no other aneuploids were detected in the larger progeny set (Figure 9A). Further PCR and RFLP tests confirmed that these F1 progeny strains were products of meiosis (Figure S12).

In addition, as a previous study showed that the *SXI2* **a** homeodomain factor gene is sufficient to drive sexual development of haploid α cells [56], we introduced the *SXI2* **a** gene into strain XL280α to mimic **a**-α sexual reproduction, generating strain MN140.23 (Figure S13). Among 90 isolates generated from the selfing of this α+*SXI2* **a** self-fertile strain, seven strains exhibited phenotypic changes compared to the parental strain (Figure S14). Further CGH analysis showed that two of these strains were aneuploid (Figure 9B). These results provide evidence that aneuploidy is also generated by **a**-α sexual reproduction.

To further examine this hypothesis, we analyzed **a**-α progeny from the cross between the isogenic *C. neoformans* var. *grubii* strains KN99**a** and KN99α. Among 88 progeny, 14 strains exhibited phenotypic changes (Figure S15), and eight were aneuploid (Figure 9C), indicating that **a**-α sexual reproduction between isogenic strains in the serotype A lineage can also generate phenotypic and genotypic diversity frequently involving aneuploid progeny. In summary, aneuploidy is generated during both α-α unisexual and **a**-α congenic sexual reproduction in both *C. neoformans* var. *grubii* (serotype A) and *C. neoformans* var. *neoformans* (serotype D).

## Discussion

While sexual reproduction serves to admix genetic diversity from two distinct parents to produce progeny with a diverse genetic repertoire, sex also comes with a series of costs. Such costs include: (1) metabolic energy that must be devoted to mating and meiosis; (2) energy and time expended locating a mating partner; (3) that only 50% of parental genes are transmitted to any given progeny or that two individuals are required to produce one progeny (resulting in the so-called 2-fold cost of sex); and (4) the fact that two genomes that have run the gauntlet of adaptive selection are shuffled during the process, breaking apart well-adapted genomic configurations [57]. A central question then is: Why would an organism engage in unisexual reproduction? In some examples, unisex involves two genetically distinct α mating partners, and this leads to genetic exchange and the production of recombinant progeny, similar to **a**-α sexual reproduction. Also, α-α mating lowers the barrier to finding a rare mate in a predominantly α population, enabling outcrossing even if no **a** partners are available. But in other cases, an α isolate undergoes unisexual reproduction all by itself (via cell–cell fusion or endoreplication), and in these cases *there is no pre-existing genetic diversity to admix*. If there is no heterozygosity of the diploid undergoing meiosis, why undergo sex if the genome is homozygous everywhere?

As shown here, our studies provide direct experimental support for the hypothesis that unisexual reproduction can generate phenotypic and genotypic diversity *de novo*. Why might this be of selective and adaptive benefit? In the context of considering the costs of sexual reproduction, unisexual reproduction is one strategy by which several of the costs normally associated with sex can be lessened or mitigated entirely. First, the cost of finding a mating partner is considerably decreased in the case of mother–daughter cell mating in which the two cells are physically juxtaposed or eliminated entirely, such as in endoreplication, in which a single cell transitions from haploid to diploid as a prelude to meiosis. Second, during selfing unisexual reproduction, ∼100% of the parental genes are directly transmitted to the F1 progeny, thus reducing the 2-fold cost of sex. Further, if one considers aneuploidy, >100% of the parental genes are transmitted to progeny. Third, unisexual reproduction eliminates the cost of sex associated with breaking apart well-adapted genomic configurations. Instead, unisexual reproduction preserves well-adapted genotypes by allowing mating between genetically identical cells (i.e., mother and daughter cells) and adding a limited amount of genetic diversity (including aneuploidy, chromosomal size polymorphisms/deletions, and SNPs) to a well-adapted genotype. In essence, unisexual reproduction provides a mechanism by which a well-adapted genotype can be changed much more subtly than standard sexual outcrossing, and in the case of well-adapted genotypes has the capacity to provide a more parsimonious route to progeny that are enhanced in competitive fitness in response to subtle changes in environmental conditions.

Organisms that reproduce sexually have an advantage over organisms that reproduce asexually, as preexisting genetic diversity will generate novel combinations in the population. Meiosis, through homologous recombination, will generate distinct genetic compositions that may have a selective advantage over the parents in a new hostile environment. Unisexual reproduction is a sexual cycle that can occur between genetically identical cells and, as we observed here, allows the introduction of limited *de novo* genetic diversity through meiosis. As in most species, meiosis in *Cryptococcus* is a highly regulated process. Comparative genomics reveals that *Cryptococcus* species contain the meiotic genes involved in homologous recombination and synaptonemal complex formation [46],[58]. The genes that support the presence of a meiotic pathway (termed the meiotic toolkit genes: *DMC1*, *MND1*, *MSH4*, *MSH5*, *SPO11*, *HOP1*, *HOP2*, and *REC8*) are highly conserved in *Cryptococcus*, and two of these (*SPO11* and *DMC1*) have been found to be critical for sporulation and spore germination during unisexual reproduction [34],[42].

Sex-induced phenotypic and genotypic variation in a clonal population is not restricted to the *Cryptococcus* genus. *Aspergillus nidulans* is a filamentous ascomycete that, along with its well-established sexual cycle, also has a parasexual cycle wherein haploid mycelia fuse and then their nuclei fuse to form diploid nuclei [59]. In the parasexual cycle, the diploid mycelium undergoes a transient aneuploid state by repeated loss of whole chromosomes to ultimately regenerate haploid progeny. A recent study has shown that the *A. nidulans* parasexual cycle can drive adaptive evolution. Hoekstra and colleagues generated homozygous diploid strains that were isogenic with their haploid progenitor [60]. In a laboratory setting, faster growing variants frequently arose from the homozygous diploid strains but not from their haploid parents. Remarkably, all of the faster growing variants derived from a diploid parent were found to be haploids that arose through the parasexual cycle. As few as 3,000 generations were sufficient for the emergence of more rapid growth. Through genetic analysis, this study supported a model wherein the diploid serves as a capacitator for evolution. This enables recessive mutations to arise sequentially in the sheltered state of the diploid where they are complemented, and these mutations do not survive in the haploid because they are individually deleterious and swept from the population before a second mutation can arise [60]. The parasexual state assorts and releases these mutations into the haploid state, where they exhibit reverse epistasis and only when in combination confer a benefit (i.e., faster growth), whereas individually each recessive mutation was deleterious. If parasexual cycles can function as capacitators to generate, store, and then release genotypic and phenotypic diversity *de novo*, we propose that sexual cycles, including unisexual reproduction, might also serve such a role in which meiosis rather than parasexual chromosome loss is involved.

A similar unisexual selfing mechanism has been observed in the human fungal pathogen *C. albicans*. Alby et al. found that **a**/**a** cells of *C. albicans* lacking the Bar1 protease that destroys the α-factor mating pheromone undergo **a**/**a**-**a**/**a** unisexual mating, yielding tetraploids that can undergo concerted chromosome loss to complete a parasexual cycle [43]. In addition, the parasexual cycle is induced between two **a**/**a** mating partners when mixed in ménage a trois matings with a limited number of α/α cells that serve as pheromone donors to trigger unisexual **a**-**a** reproduction [43]. However, the parasexual progeny of *C. albicans* are characterized by high rates of aneuploidy [44]. Although this chromosomal assortment process is imprecise, it may be retained due to a selective pressure that favors aneuploid strains under certain environmental conditions, such as in patients receiving fluconazole, a situation in which an isochromosome 5 derivative that confers drug resistance often arises [15]. Aneuploidy has been linked to phenotypic changes, including resistance to antifungal drugs in both *C. albicans* and *C. neoformans*, which could provide a selective advantage during infection [15]–[17].

The positive impact of aneuploidy on evolution and genetic diversification extends beyond the fungal kingdom to other unicellular eukaryotes. The parasitic protozoan *Leishmania* is the etiological agent behind one of the most common neglected diseases, leishmaniasis, a global cause of morbidity and mortality. No vaccine is available, and treatment relies heavily on pentavalent antimonial compounds with diminishing efficacy due to emerging drug resistance [61]. Recent studies have shown that aneuploidy is widespread in natural and clinical populations, with variation in the ploidy state (monosomic, disomic, or trisomic) for different chromosomes even within the same isolate [62],[63]. Mosaic aneuploidy in *Leishmania* generates dynamic genome plasticity, is well tolerated, and can confer drug resistance [22],[62], analogous to fungal azole resistance conferred by aneuploidy.

Given the finding that aneuploidy can underlie and drive adaptive evolution in *S. cerevisiae*, *C. albicans*, *C. neoformans*, and *Leishmania*, it seems likely that beneficial impacts of aneuploidy may be even more ubiquitous and remain to be discovered in other saprobic and pathogenic eukaryotic microbes.

## Materials and Methods

### Ethics Statement

All of the animal studies were conducted in the Division of Laboratory Animal Resources (DLAR) facilities at Duke University Medical Center (DUMC). All of the animal work was performed according to the guidelines of NIH and Duke University Institutional Animal Care and Use Committee (IACUC). The animal experiments were reviewed and approved by the DUMC IACUC under protocol number A266-08-10.

### Strains and Media

Strains and plasmids used in this study are listed in Table S4. Yeast cells were grown on YPD media. Mating of *C. neoformans* was conducted on 5% V8 juice agar medium (pH = 7).

### Sequencing of *C. neoformans*

DNA sequencing was performed on a Genome Analyzer IIx using Illumina Paired Ends technology. The genomic DNA samples were fragmented with a Bioruptor sonicator (Diagenode). Fragmented DNA was used for size selection by extraction (Qiagen) of 300 to 400 bp DNA fragments from 2% agarose gels after electrophoresis. Size-selected DNA was used for standard Illumina library preparation protocols. Prepared libraries were sequenced using Paired Ends 2×76 cycles. This approach provided the most suitable sequencing data for SNP and small indel detection and deep sequencing coverage.

### Genomic Sequence Assembly Protocol

The Illumina-Solexa data were assembled using a combination of *de novo* assembly (Velvet) and reference genome assembly (BWA) to assemble the *Cryptococcus* genomic sequence. We used BWA and SAMtools to determine the depth of sequence coverage and identify polymorphisms, particularly SNPs and indels. In addition to the use of BWA for reference genome assembly, we checked the completeness of the assemblies by extracting all sequence reads that BWA was unable to align to the reference genome and assembled these reads using Velvet. The resulting contigs were compared back to the genome sequence and also used to search GenBank using Blast. This method allows identification of regions with multiple polymorphisms that cannot be identified by BWA due to the inability of the short reads to be aligned to the reference sequence. In addition, these contigs typically originate from highly repetitive sequence, such as the rDNA and telomeres. With this *Cryptococcus* dataset, more than 90% of the unaligned reads were low-quality sequence reads. Overall, the assembly was an iterative process. After polymorphisms were identified in an initial round of sequence assembly with BWA and Velvet, a new consensus sequence was generated, and the assembly was repeated. In the second and subsequent rounds of assembly, polymorphisms were identified in regions where there were multiple polymorphisms. After several rounds, no additional polymorphisms were identified, and the assembly was considered complete (Figure S1).

### Ploidy Determination by FACS

The ploidy of progeny was determined by flow cytometry as described previously [64]. Briefly, cells were collected from overnight YPD liquid medium, washed once in 1× PBS buffer, and fixed in 1 mL of 70% ethanol overnight at 4°C. Fixed cells were washed once with 1 mL of NS buffer (10 mM Tris-HCl [pH = 7.6]; 250 mM sucrose; 1 mM EDTA [pH = 8]; 1 mM MgCl_2_; 0.1 mM CaCl_2_; and 0.1 mM ZnCl_2_) and then stained with propidium iodide (0.3 mg/mL) in 200 µL of NS buffer containing RNaseA (1 mg/mL) at room temperature for 4 h. Then, 50 µL of the stained cell preparation was diluted into 2 mL of 50 mM Tris-HCl (pH = 7.5) and sonicated for 1 min. Flow cytometry was performed on 10,000 cells and analyzed on the FL1 channel of a Becton-Dickinson FACScan.

### Complementation of *hsc20-1* TS Mutation in MN7

The strain MN7 was transformed with the dominant selectable drug marker NEO, amplified from plasmid pJAF1 using the universal primers M13F and M13R. The strain MN7 *NEO* was mixed with strain XL280α *NAT* and incubated on mating media for 48 h. The mating culture was scraped off the plate, washed in water, serially diluted, and plated on YPD+NAT+NEO medium. The ploidy of the fusion products was determined by FACS. Multiple diploid isolates were phenotypically analyzed at 30°C and 37°C to determine the recessive nature of the TS mutation in strain MN7.

The wild-type *HSC20* gene with its native promoter (269 bp) and terminator (246 bp) was amplified from XL280α genomic DNA using the primer pair JOHE38840/JOHE38841 and cloned in the pJAF12 plasmid using KpnI restriction enzyme (NEB) [65]. Two independently derived plasmids, pMF86 and pMF89, were obtained and confirmed by sequencing. The plasmids were introduced into strain MN7 via biolistic transformation, and multiple isolates were obtained and analyzed phenotypically.

### Pulsed-Field Gel Electrophoresis (PFGE) of *C. neoformans*

A single colony was inoculated into 5 mL of liquid YPD medium and grown overnight at room temperature (RT) while shaking. Then, 1 mL of the culture was added to 50 mL of yeast nitrogen base minimal medium (YNB) with 1 M NaCl (to suppress capsule formation), which was then grown overnight at RT on a shaking platform incubator. Yeast cells were then washed three times in 0.5 M NaCl/50 mM EDTA (pH = 8.0). Then, 50 mg of cells were mixed with 450 µL of low-melting point agarose (Bio-Rad 0.5% in 0.1 M EDTA [pH = 8.0]) and 20 µL of cell wall lysing buffer (25 mg/mL Zymolase in 10 mM KPO_4_ [pH = 7.5]) and poured into molds. The plugs were solidified for 15 min at RT, transferred into 700 µL of lysing solution (0.5 M EDTA, 10 mM Tris-HCl [pH = 8]), and lysed overnight at 37°C. The next morning, 400 µL of proteinase K solution (5% sarcosyl, 5 mg/ml proteinase K, 0.5 M EDTA [pH = 8.0]) were added, and the plugs were further incubated at 50°C for 5 h. The plugs were then washed three times for 1 h each with wash solution (50 mM EDTA, 10 mM Tris-HCl [pH = 8.0]). Then, 1% gels made with pulse field certified agarose in 0.5× TBE buffer were run with a BioRad CHEF Mapper XA system. Running conditions were according to the CHEF Mapper calculation, with appropriate modifications.

### CGH and Band Array

Genomic DNA was ultrasonicated to generate ∼500-bp fragments and purified with a DNA Clean and Concentrator kit (Zymo Research, CA). Then, 2.5 µg of DNA was used for Cy3 dUTP or Cy5 dUTP labeling reactions using the Random Primer/Reaction Buffer mix (BioPrime Array CGH Genomic Labeling System, Invitrogen). Labeled DNA was then hybridized to microarray slides of 70-mer oligonucleotides for the *C. neoformans* JEC21 [49] or the *C. neoformans* JEC21 and H99 genomes (Washington University, St. Louis, MO). After hybridization, arrays were scanned with a GenePix 4000B scanner (Axon Instruments). Data analysis was performed with Genespring GX v7.3 (Agilent Technologies) and CGH-miner. Band array was performed as described previously [66]. Briefly, chromosomes were separated by PFGE and the bands of interest were excised and treated to extract the DNA. Band DNA was labeled with Cy5 and hybridized to microarrays with Cy3-labeled whole genome DNA.

### Multiplex PCR

Genomic DNA was extracted using the CTAB protocol as described previously [67]. We utilized the Qiagen multiplex PCR kit for multiplex PCR. PCR reaction mixtures (25 µL) contained 12.5 µL of Qiagen multiplex PCR master mix, 2 µM equimolar primer mixture (Table S3), and 100 to 300 ng of genomic DNA. Thermal cycling conditions included an initial heat activation of 15 min at 95°C followed by 30 cycles of denaturation for 30 s at 94°C, annealing for 90 s at 58.7°C, and extension for 10 min at 72°C. For bioanalyzer analysis, 1 µL of PCR reaction was examined with the Experion DNA 12K Analysis Kit (Bio-Rad). Multiplex PCR reactions were analyzed by gel electrophoresis using 1.8% agarose gels in 1×TBE buffer (Tris/Borate/EDTA buffer). 2 µL of PCR reaction were loaded in the gels that were run overnight at 30 volts or for 5 h at 100 volts. The intensity of the desired bands, which represent the aneuploid chromosomes, was quantified relative to the control band using the Gel Doc XR+ system of BIO-RAD and Image Lab version 4 software.

### Restriction Fragment Length Polymorphism (RFLP)

RFLP analysis was performed as described previously [68]. Based on the SNPs in XL280 and JEC20, we designed two RFLP markers, RFLP3 and RFLP7 (primers are shown in Table S5), and digested with NdeI and XbaI, respectively. Other markers included the *STE20* **a** and *STE20*α mating type locus genes to assign the mating type as **a** or α.

### Virulence Studies

Virulence assays were conducted using a murine inhalation model of cryptococcosis. Cohorts of 4- to 8-wk-old female DBA mice were anesthetized through intraperitoneal injection of Nembutal (37.5 mg/kg) and infected intranasally with 5×10^6^ cells diluted in sterile PBS. The cells of the inoculum were diluted and plated onto YPD medium to determine CFU and viability. Mice were monitored twice daily, and moribund individuals were euthanized with CO_2_. The survival rates were plotted against time using Kaplan-Meier survival curves, generated with Prism 4.0 (GraphPad software, La Jolla, CA, USA). The *p* values were evaluated by a Log-rank (Mantel-Cox) test. A *p* value of <0.05 was considered significant. The lungs and brains of the euthanized animals were removed, weighed, and homogenized in 2 ml sterile PBS. The samples were serial diluted and plated on YPD media to count CFUs. Twenty random isolates were colony purified and subjected to multiplex PCR to detect aneuploidy.

### Competitive Fitness and Growth Curve Assays

XL280, MN55, MN77, MN89 (all NAT sensitive), and XL280 marked with the NAT resistance marker integrated at the *SPO11* genetic locus (XL280 *NAT*) were used to measure the competitive fitness of the aneuploid strains in the presence of fluconazole. Strains were grown overnight in liquid cultures in YPD, washed with sterile water, and diluted to a density of 1×10^7^ cells/ml. The following strains were mixed in equal ratios: XL280 with XL280 *NAT*, MN55 with XL280 *NAT*, MN77 with XL280 *NAT*, and MN89 with XL280 *NAT*. The mixtures were spotted on YPD and YPD plus 8 µg/mL fluconazole (FLC) and incubated for 3 d at 30°C. Then, the cells were recovered from the plates, washed with sterile water, and plated on YPD to count CFUs. Following 2 d incubation at 30°C, 300 isolates were replica plated onto NAT selective media and survival rates were calculated. The experiments were conducted in triplicate. For the growth curve assays, the strains were grown overnight in liquid cultures, washed with sterile water, and diluted into 4×10^4^ cells in 200 µl of YPD and YPD plus 2 µg/mL fluconazole (fluconazole concentration was determined experimentally). The cells were incubated at room temperature in 96-well plates, and OD_600_ was measured every 2 h for 48 h using a Tecan-Sunrise microplate reader. All of the experiments were performed in triplicate.

## Supporting Information

Figure S1 **NGS data analysis pipeline.** Approximately 48 million 75 bp reads were aligned to the reference genome (JEC21) representing ∼160× coverage. By performing BWA and SAM tools analysis, we detected SNPs and small indels. Unaligned reads were assembled *de novo* by Velvet analysis and blasted against the reference genome. The new genome was assembled by replacing SNPs. The reads were again aligned to newly assembled genome assemblies to further detect any SNPs that escaped the previous round of analysis. The whole cycle was repeated 30 times to assemble the XL280 genome.
(TIF)

Figure S2 **Band array data for XL280 chromosomes.** DNA of each band (Chr 5, Chr 6/7, Chr 8/9, Chr 10, Chr 11, Chr 12, Chr 13, and Chr 14) was extracted from the PFGE gel (A) and analyzed by CGH (B). Coloring indicates gene dosage as follows: gray, no significant change; red, more abundant; green, less abundant. Sc represents the 0.225–2.2 Mb *S. cerevisiae* CHEF DNA Size Markers (BioRad 170-3605).
(TIF)

Figure S3 **Unisexual reproduction progeny of XL280 exhibit novel phenotypes.** Strains were spotted in 10-fold serial dilutions and grown under the following conditions: (A) YPD at 30°C for 2 d, (B) YPD at 37°C for 2 d, and (C) YPD plus 8 µg/mL fluconazole (FLC) at 30°C for 4 d. Red labels indicate the progeny with variant phenotypes compared to the parental strain.
(TIF)

Figure S4 **Proliferative capacity of aneuploid strains in the presence of drugs or chemicals.** Aneuploid strains were 10-fold serially diluted, spotted, and grown under the following conditions: (A) YPD at 30°C for 2 d, (B) YPD plus 10 µg/mL hygromycin at 30°C for 2 d, (C) YPD plus 0.1 µg/mL cycloheximide at 30°C for 3 d, (D) YPD plus 0.1 µg/mL cycloheximide at 30°C for 4 d, (E) YPD plus 200 mM rapamycin at 30°C for 3 d; (F) YPD plus 0.1 mM benomyl at 30°C for 2 d; and (G) YPD plus 0.5 mM H_2_O_2_ at 30°C for 4 d.
(TIF)

Figure S5 **XL280 mitotic progeny grown on YPD did not exhibit any phenotypic changes.** Strains were 10-fold serially diluted, spotted, and grown under the following conditions: (A) YPD at 30°C for 2 d, (B) YPD at 37°C for 2 d, and (C) YPD plus 8 µg/mL fluconazole (FLC) at 30°C for 4 d. In no case was any progeny produced by mitosis found to differ phenotypically from the XL280 parental strain.
(TIF)

Figure S6 **Bioanalyzer profiles of multiplex PCR reactions.** Genomic DNA from XL280 (A, control strain) and aneuploid progeny MN77 (n+1^10^) (B) served as template. PCR products were analyzed via the BioRad Experion Bioanalyzer System, which identified amplicons that differed in abundance between the strains (blue arrows).
(TIF)

Figure S7 **Multiplex PCR on XL280 meiotic progeny.** Genomic DNA from 90 unisexual reproduction meiotic progeny was isolated and subjected to multiplex PCR to detect aneuploidy. All four aneuploid strains are marked with red. All other 86 progeny were found to be euploids in two replicates of this analysis.
(TIF)

Figure S8 **Multiplex PCR on XL280 mitotic progeny.** Ninety-six isolates of XL280 derived from mitotic asexual growth on YPD were selected. DNA was isolated and subjected to multiplex PCR to search for aneuploidy. No aneuploids were detected among the 96 progeny, which were all euploid.
(TIF)

Figure S9 **Aneuploid strains were stable inside the murine host.** Cells were isolated from homogenized lungs of infected animals with MN35 (n+1^13^), MN55 (n+1^9^), MN77 (n+1^10^), and MN89 (n+1^10^), and DNA was isolated from 20 colony-purified isolates and subjected to multiplex PCR to detect aneuploidy. The abundance of the aneuploid/euploid chromosome was quantified by estimating the relative intensity of the desired chromosomal bands compared to the respective control. Aneuploid strains are marked with red, whereas strains that reverted to euploid are marked black.
(TIF)

Figure S10 **Aneuploidy also causes the observed phenotypic changes in MN35 and MN55.** MN35 (n+1^13^) and MN55 (n+1^9^) were grown on YPD for 3 d to promote the loss of the extra chromosome. Twenty isolates were picked and they were subjected to phenotypic and genotypic analysis. (A) MN35 (n+1^13^) and 20 isolates were grown, 10-fold serially diluted, and spotted on YPD at 30°C for 2 d, YPD at 37°C for 2 d, and YPD plus 8 µg/mL fluconazole (FLC) at 30°C for 4 d. Multiplex PCR detect an extra Chr 13 in all of the strains with the fluconazole sensitivity observed in MN35. (B) The MN55 (n+1^9^) and 20 isolates were spotted in 10-fold serial dilutions and incubated under the same conditions. Multiplex PCR confirmed that the isolates with the fluconazole resistant phenotype carry an extra chromosome 9.
(TIF)

Figure S11 **Opposite sexual reproduction progeny of XL280 crossed with JEC20 exhibit phenotypic variation.** Strains were spotted in 10-fold serial dilutions and grown under the following conditions: (A) YPD at 30°C for 2 d, (B) YPD at 37°C for 2 d, (C) YPD plus 8 µg/mL fluconazole (FLC) at 30°C for 4 d, and (D) YPD plus 1 µg/mL FK506 at 30°C for 2 d. α and **a** represent the parental strains XL280 and JEC20, respectively. Red labels indicate the progeny whose phenotypes differed compared to the parental strains.
(TIF)

Figure S12 **Aneuploid strains from the cross between XL280 and JEC20 are recombinants.** Four markers were analyzed: *STE20*α, *STE20* **a**, RFLP3, and RFLP7. MM10, MM16, MM51, and MM67 are four aneuploid progeny from the cross between strains XL280 and JEC20.
(TIF)

Figure S13 ***SXI2*** **a is overexpressed in MN140.23.** Northern blot analysis using the *SXI2* **a** gene as a probe detected the overexpressed *SXI2* **a** gene in strain MN140.23, which contains a P*_GPD1_*::*SXI2* **a** transgene in the XL280 background. EtBr staining of rRNA served as a loading control.
(TIF)

Figure S14 **Unisexual reproduction progeny of MN140.23 (XL280, P** ***_GPD1_*** **::** ***SXI2*** **a) exhibit phenotypic variation.** Strains were spotted in 10-fold serial dilutions and grown under the following conditions: (A) YPD at 30°C for 2 d, (B) YPD at 37°C for 2 d, (C) YPD plus 8 µg/mL fluconazole (FLC) at 30°C for 4 d, and (D) YPD plus 1 µg/mL FK506 at 30°C for 2 d. C1, C2, and C3 represent strains XL280, XL566 (XL280 *ura5*), and MN140.23. Red labels indicate the progeny with phenotypes that differed compared to the parental strain.
(TIF)

Figure S15 **Opposite sexual reproduction progeny of isogenic serotype A strains KN99a and KN99α exhibit phenotypic variation.** Strains were 10-fold serially diluted, spotted, and grown under the following conditions: (A) YPD at 30°C for 2 d, (B) YPD at 37°C for 2 d, (C) YPD plus 8 µg/mL fluconazole (FLC) at 30°C for 4 d, and (D) YPD plus 1 µg/mL FK506 at 30°C for 2 d. The progeny that phenotypically differed from the wild-type is marked red.
(TIF)

Table S1 **Summary of reads from Illumina sequencing.**
(DOC)

Table S2 **XL280 versus JEC21 exonic and intronic changes.**
(XLS)

Table S3 **Primers used in the multiplex PCR assay.**
(DOC)

Table S4 **Strains and plasmids used in this study.**
(DOC)

Table S5 **Primers used in this study.**
(DOC)

## Abbreviations

CFU: colony forming unit
CGH: comparative genome hybridization
CHEF: contour-clamped homogenous electric field
Chr: Chromosome
DSBs: double strand breaks
FACS: fluorescence-activating cell sorting
FLC: fluconazole
NGS: next generation sequencing
NS: niger seed
PFGE: pulsed field gel electrophoresis
RFLP: restriction fragment length polymorphism
SNP: single-nucleotide polymorphism
TS: temperature sensitive
