# Supplementary figures and images for: Unisexual and Heterosexual Meiotic Reproduction Generate Aneuploidy and Phenotypic Diversity De Novo in the Yeast Cryptococcus neoformans
Source: PLoS Biol. 2013 Sep 10;11(9):e1001653. doi: 10.1371/journal.pbio.1001653 (PMC3769227; doi:10.1371/journal.pbio.1001653)

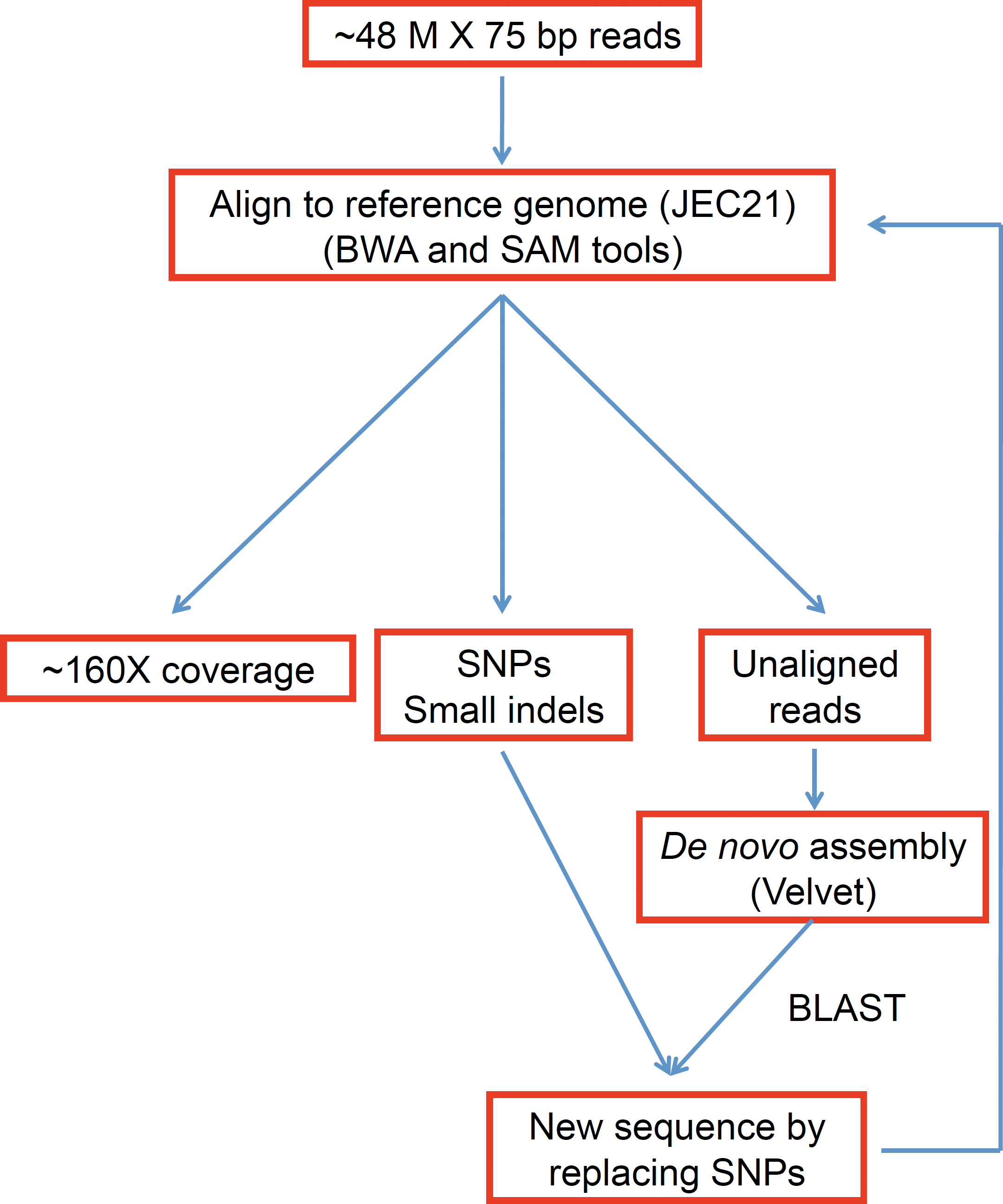

Supplement: Figure S1 — NGS data analysis pipeline. Approximately 48 million 75 bp reads were aligned to the reference genome (JEC21) representing ∼160× coverage. By performing BWA and SAM tools analysis, we detected SNPs and small indels. Unaligned reads were assembled de novo by Velvet analysis and blasted against the reference genome. The new genome was assembled by replacing SNPs. The reads were again aligned to newly assembled genome assemblies to further detect any SNPs that escaped the previous round of analysis. The whole cycle was repeated 30 times to assemble the XL280 genome. (TIF) [file pbio.1001653.s001.tif]

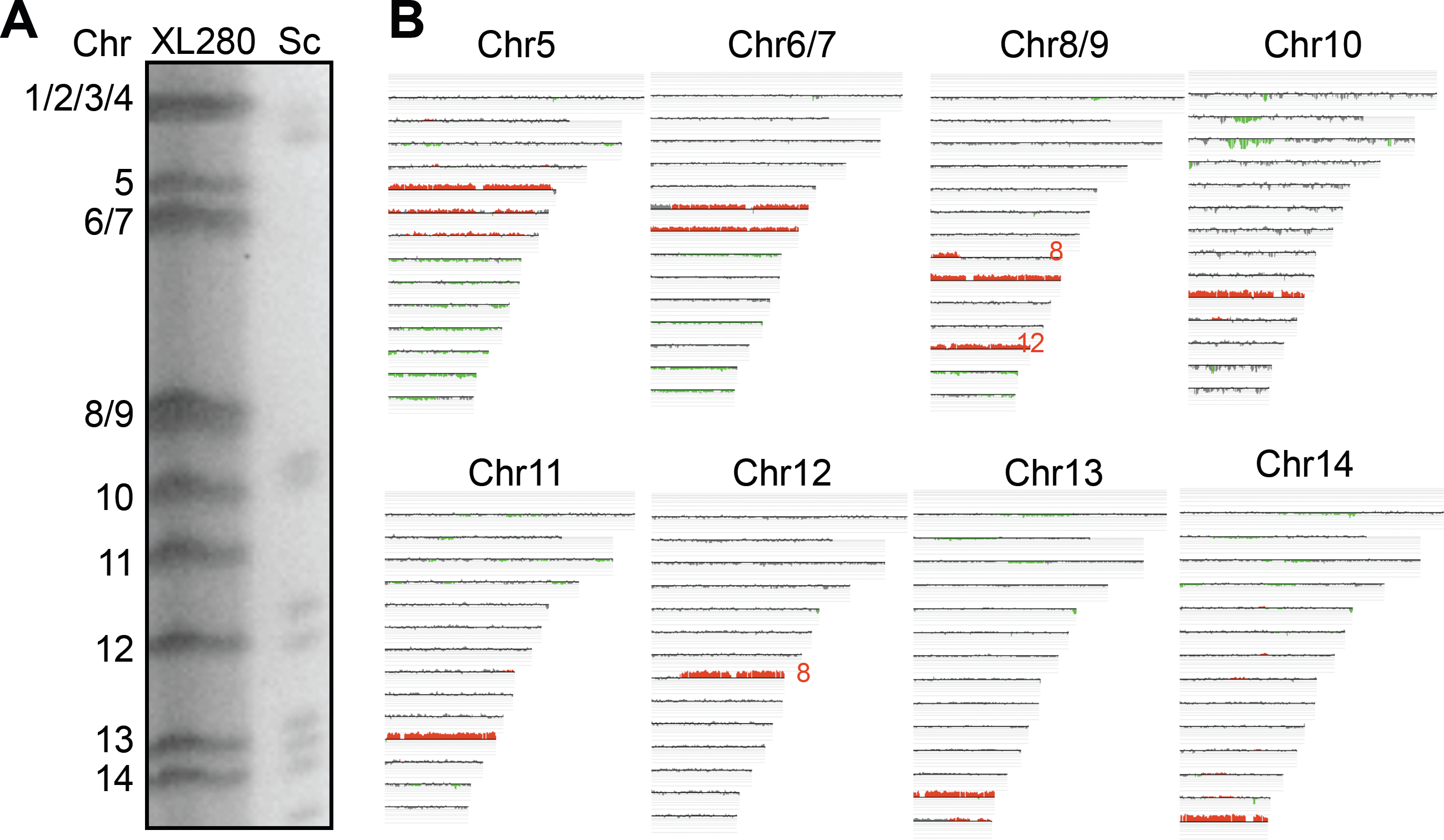

Supplement: Figure S2 — Band array data for XL280 chromosomes. DNA of each band (Chr 5, Chr 6/7, Chr 8/9, Chr 10, Chr 11, Chr 12, Chr 13, and Chr 14) was extracted from the PFGE gel (A) and analyzed by CGH (B). Coloring indicates gene dosage as follows: gray, no significant change; red, more abundant; green, less abundant. Sc represents the 0.225–2.2 Mb S. cerevisiae CHEF DNA Size Markers (BioRad 170-3605). (TIF) [file pbio.1001653.s002.tif]

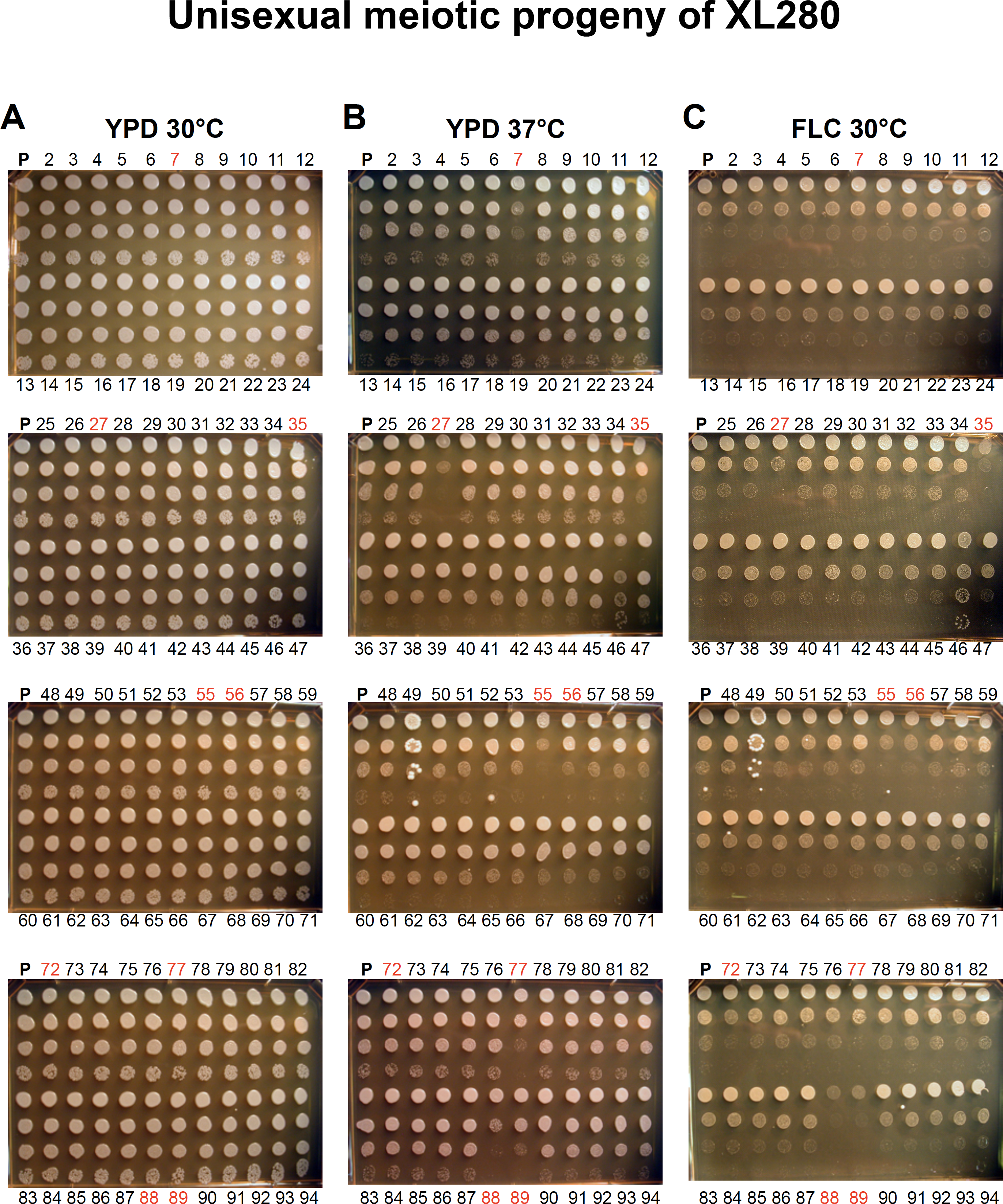

Supplement: Figure S3 — Unisexual reproduction progeny of XL280 exhibit novel phenotypes. Strains were spotted in 10-fold serial dilutions and grown under the following conditions: (A) YPD at 30°C for 2 d, (B) YPD at 37°C for 2 d, and (C) YPD plus 8 µg/mL fluconazole (FLC) at 30°C for 4 d. Red labels indicate the progeny with variant phenotypes compared to the parental strain. (TIF) [file pbio.1001653.s003.tif]

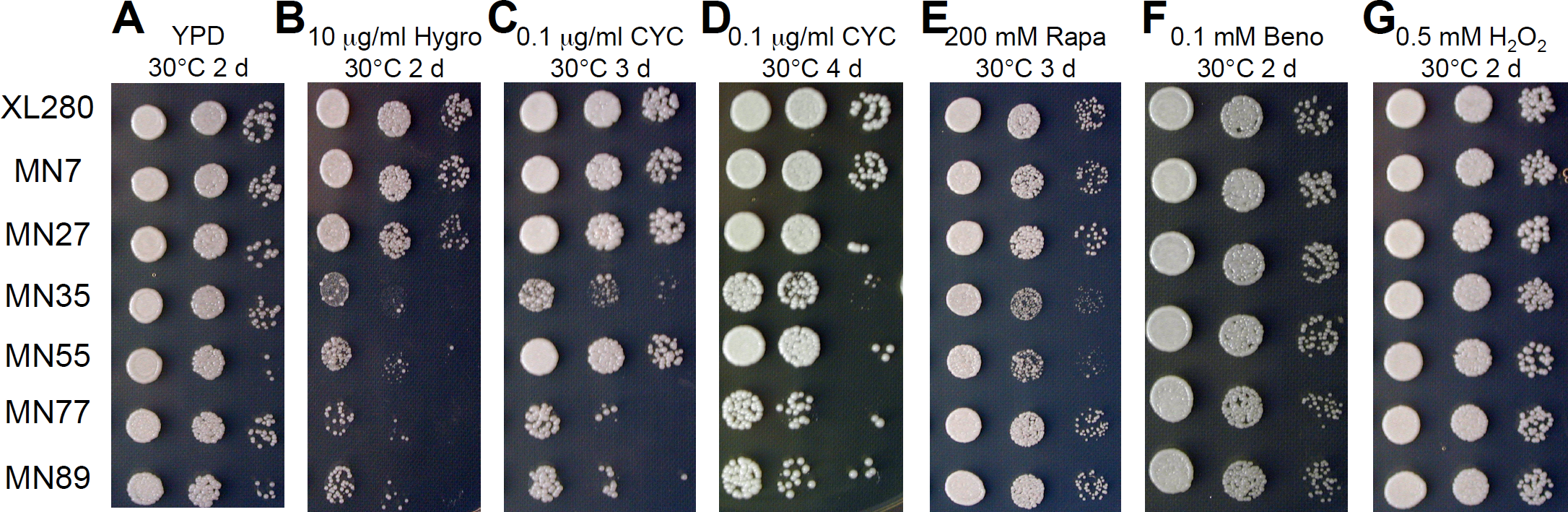

Supplement: Figure S4 — Proliferative capacity of aneuploid strains in the presence of drugs or chemicals. Aneuploid strains were 10-fold serially diluted, spotted, and grown under the following conditions: (A) YPD at 30°C for 2 d, (B) YPD plus 10 µg/mL hygromycin at 30°C for 2 d, (C) YPD plus 0.1 µg/mL cycloheximide at 30°C for 3 d, (D) YPD plus 0.1 µg/mL cycloheximide at 30°C for 4 d, (E) YPD plus 200 mM rapamycin at 30°C for 3 d; (F) YPD plus 0.1 mM benomyl at 30°C for 2 d; and (G) YPD plus 0.5 mM H2O2 at 30°C for 4 d. (TIF) [file pbio.1001653.s004.tif]

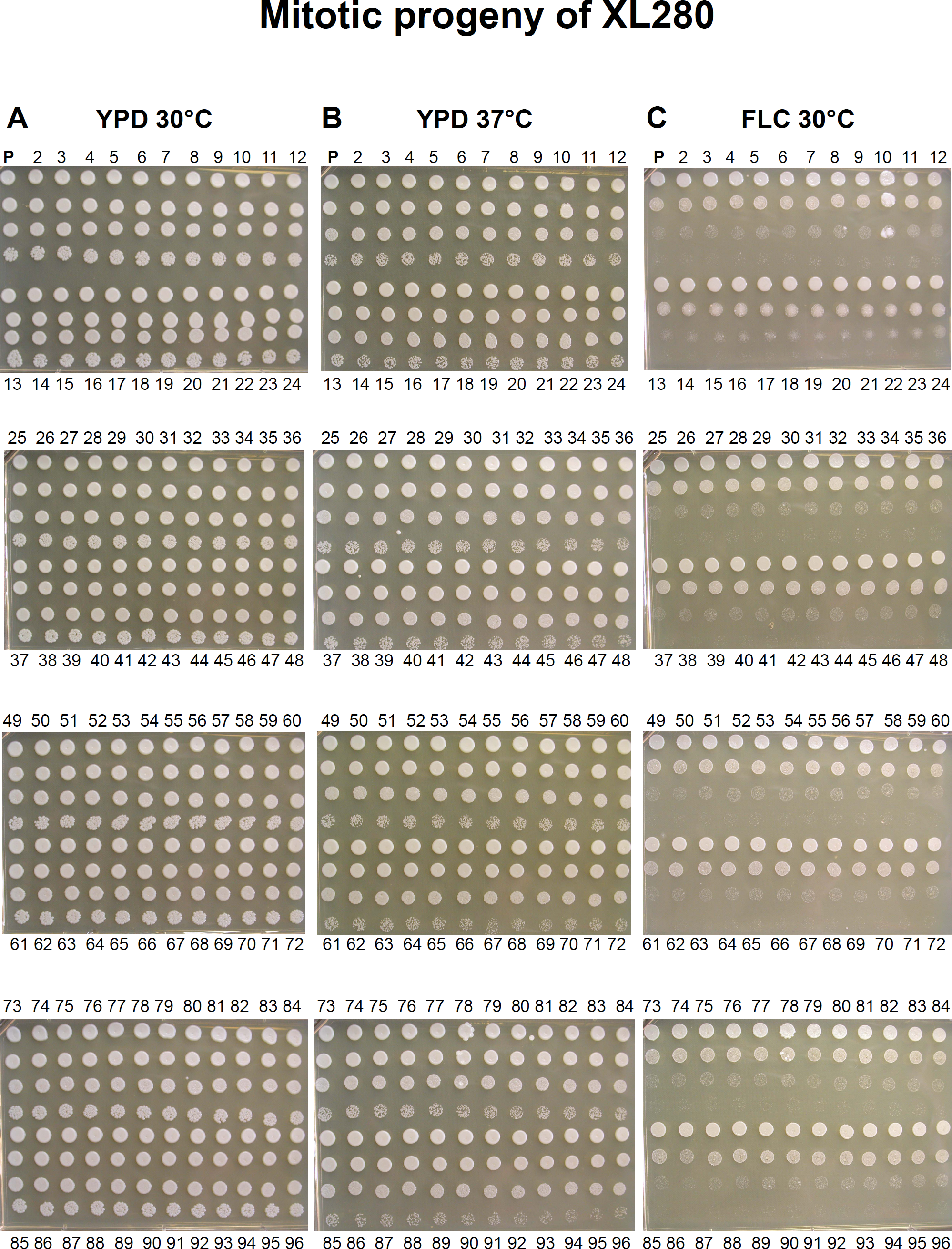

Supplement: Figure S5 — XL280 mitotic progeny grown on YPD did not exhibit any phenotypic changes. Strains were 10-fold serially diluted, spotted, and grown under the following conditions: (A) YPD at 30°C for 2 d, (B) YPD at 37°C for 2 d, and (C) YPD plus 8 µg/mL fluconazole (FLC) at 30°C for 4 d. In no case was any progeny produced by mitosis found to differ phenotypically from the XL280 parental strain. (TIF) [file pbio.1001653.s005.tif]

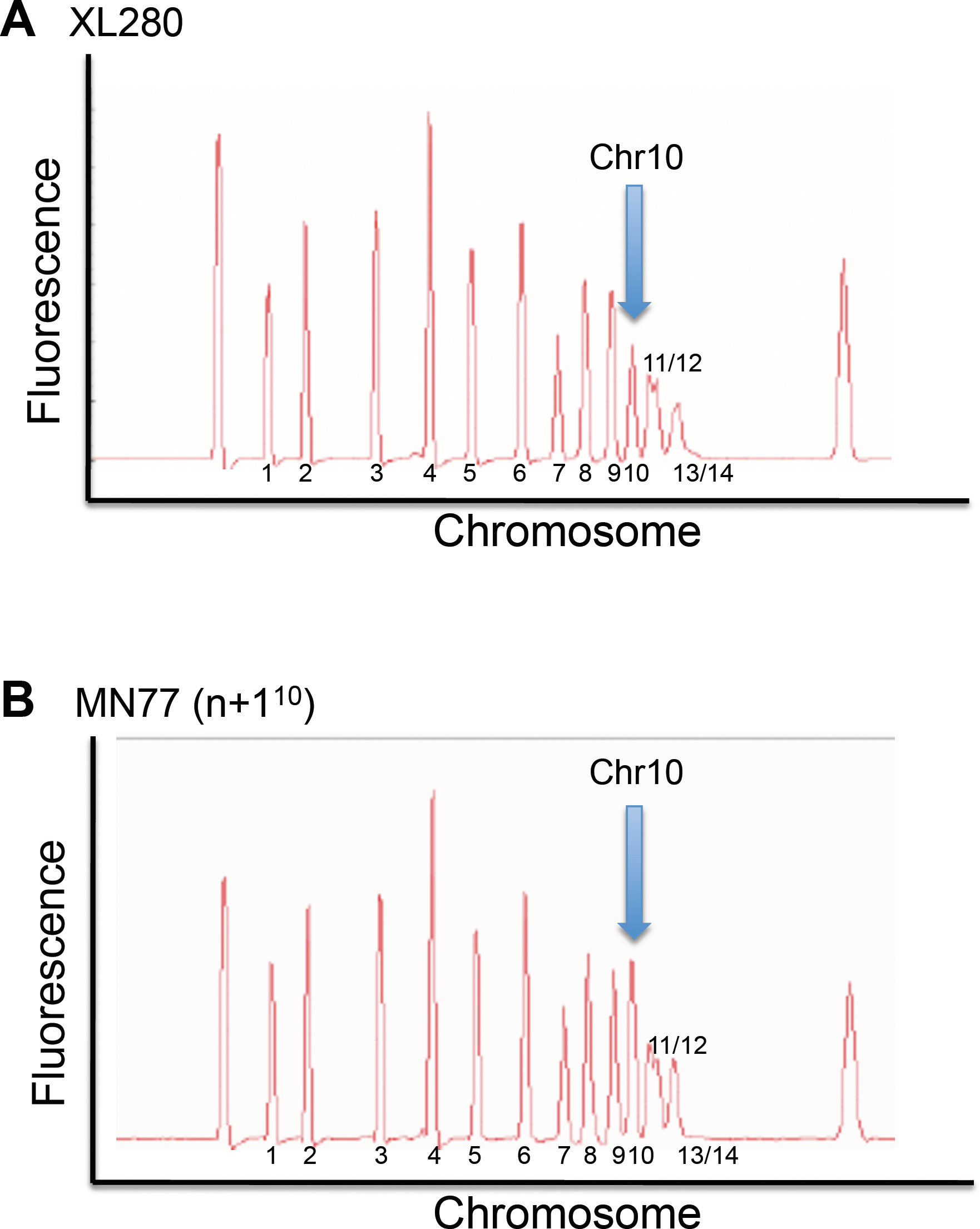

Supplement: Figure S6 — Bioanalyzer profiles of multiplex PCR reactions. Genomic DNA from XL280 (A, control strain) and aneuploid progeny MN77 (n+110) (B) served as template. PCR products were analyzed via the BioRad Experion Bioanalyzer System, which identified amplicons that differed in abundance between the strains (blue arrows). (TIF) [file pbio.1001653.s006.tif]

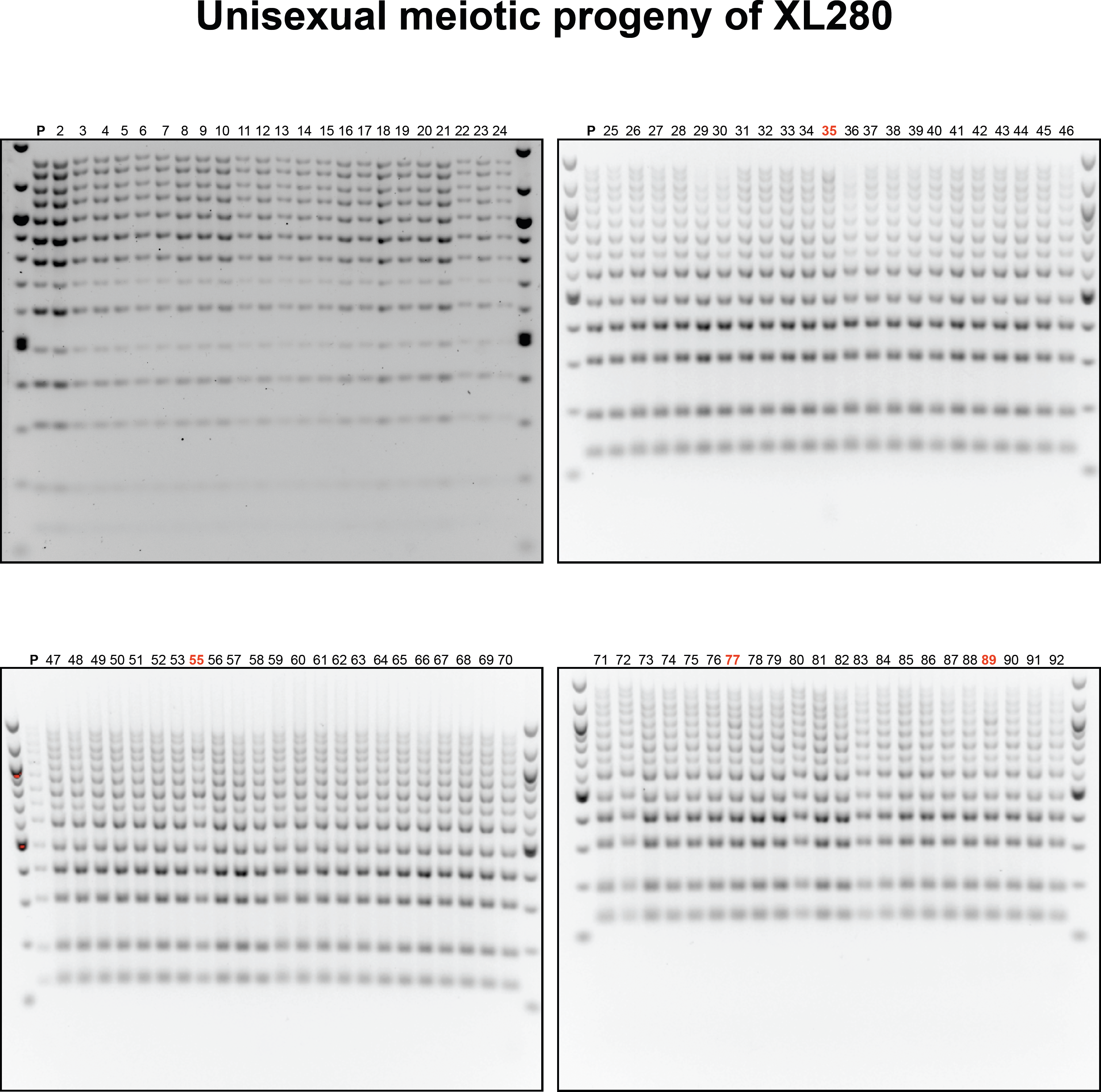

Supplement: Figure S7 — Multiplex PCR on XL280 meiotic progeny. Genomic DNA from 90 unisexual reproduction meiotic progeny was isolated and subjected to multiplex PCR to detect aneuploidy. All four aneuploid strains are marked with red. All other 86 progeny were found to be euploids in two replicates of this analysis. (TIF) [file pbio.1001653.s007.tif]

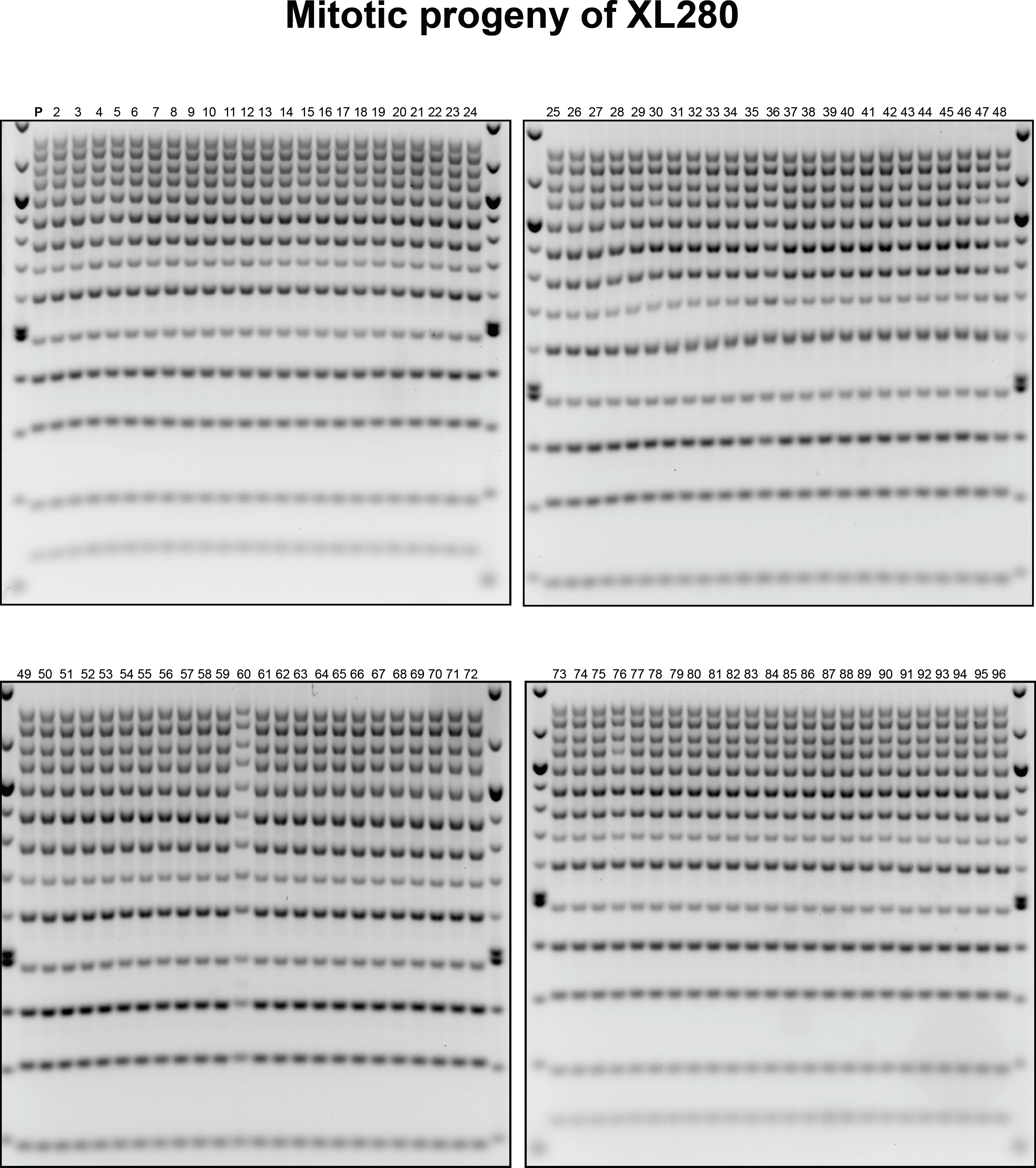

Supplement: Figure S8 — Multiplex PCR on XL280 mitotic progeny. Ninety-six isolates of XL280 derived from mitotic asexual growth on YPD were selected. DNA was isolated and subjected to multiplex PCR to search for aneuploidy. No aneuploids were detected among the 96 progeny, which were all euploid. (TIF) [file pbio.1001653.s008.tif]

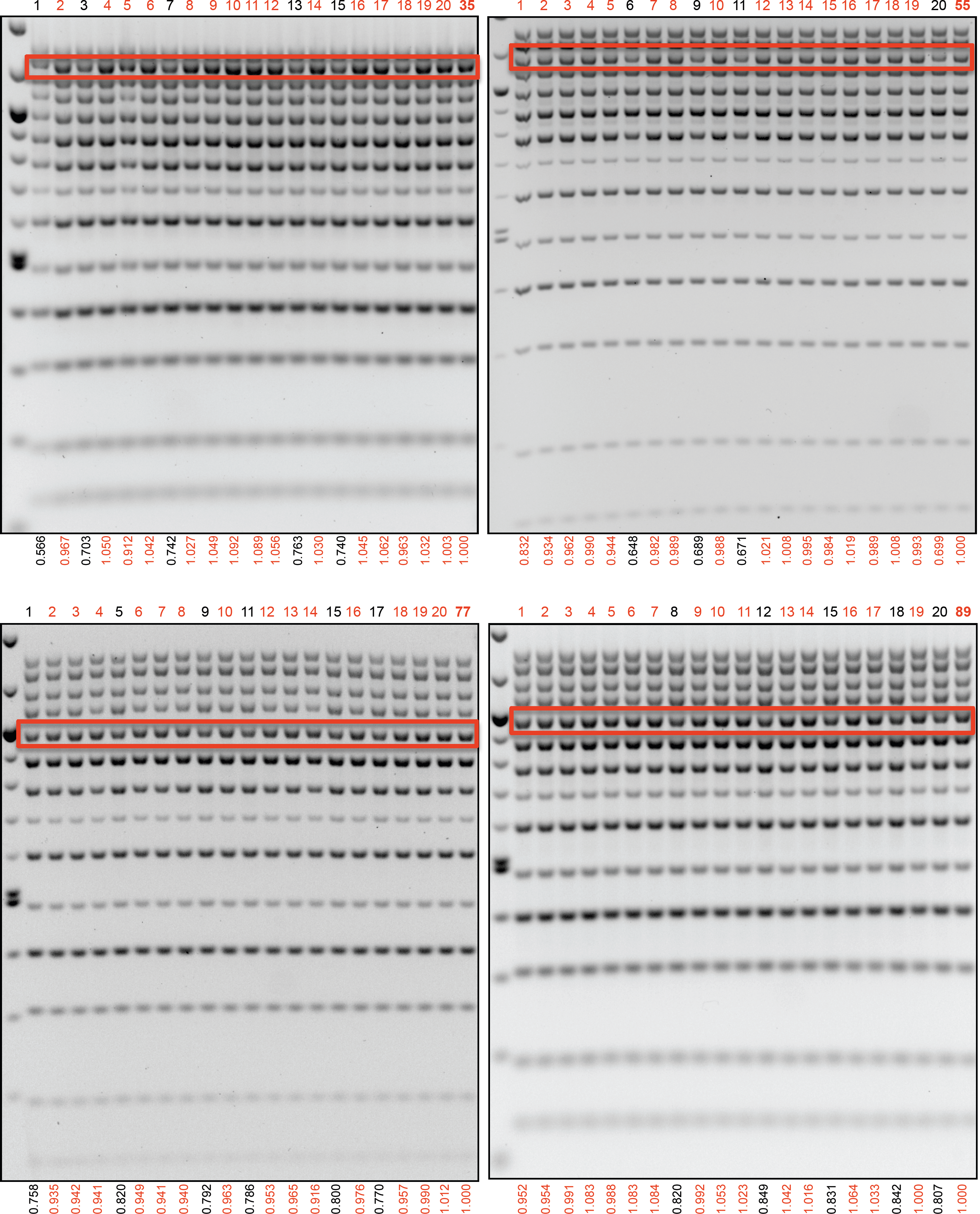

Supplement: Figure S9 — Aneuploid strains were stable inside the murine host. Cells were isolated from homogenized lungs of infected animals with MN35 (n+113), MN55 (n+19), MN77 (n+110), and MN89 (n+110), and DNA was isolated from 20 colony-purified isolates and subjected to multiplex PCR to detect aneuploidy. The abundance of the aneuploid/euploid chromosome was quantified by estimating the relative intensity of the desired chromosomal bands compared to the respective control. Aneuploid strains are marked with red, whereas strains that reverted to euploid are marked black. (TIF) [file pbio.1001653.s009.tif]

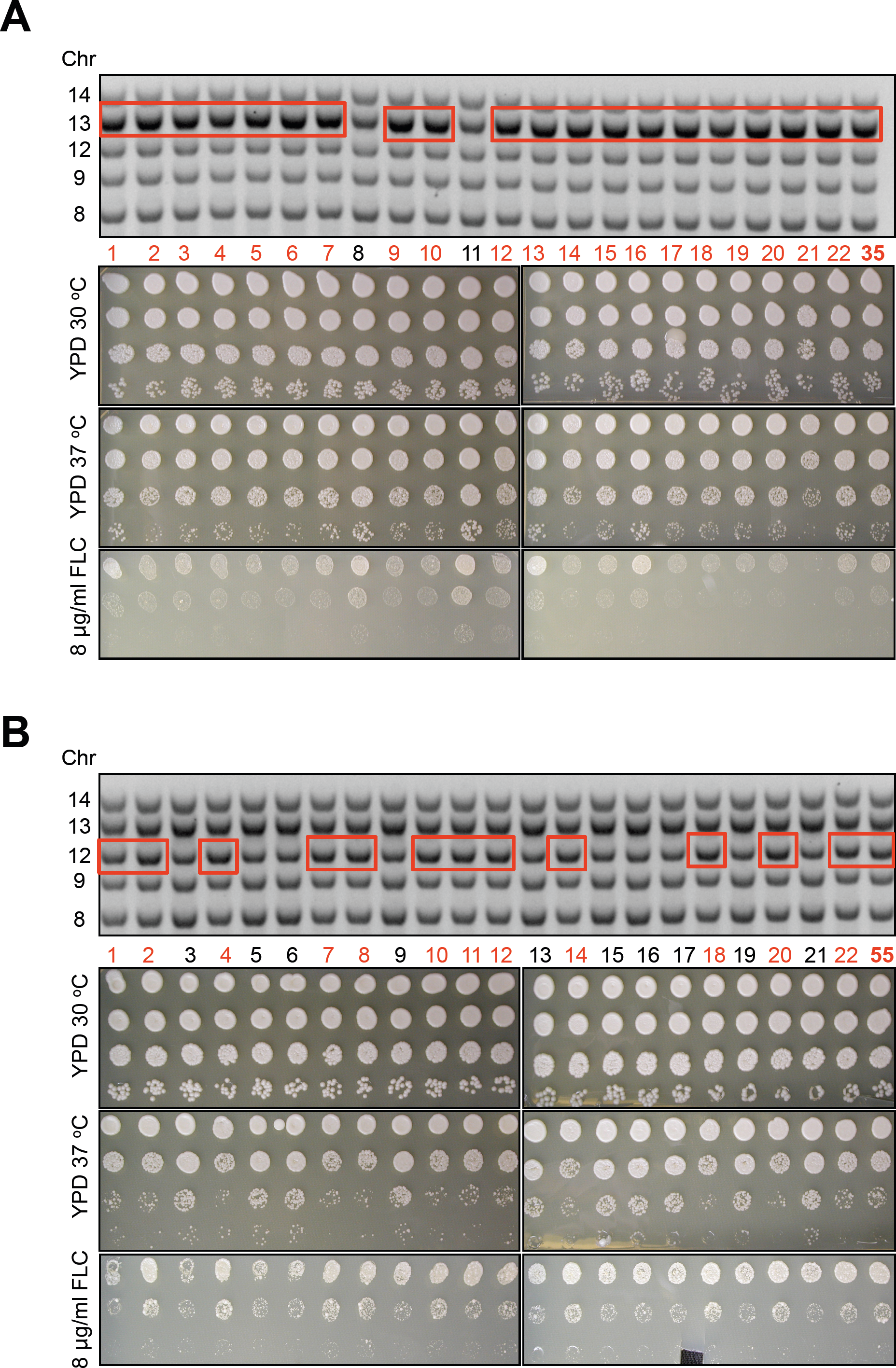

Supplement: Figure S10 — Aneuploidy also causes the observed phenotypic changes in MN35 and MN55. MN35 (n+113) and MN55 (n+19) were grown on YPD for 3 d to promote the loss of the extra chromosome. Twenty isolates were picked and they were subjected to phenotypic and genotypic analysis. (A) MN35 (n+113) and 20 isolates were grown, 10-fold serially diluted, and spotted on YPD at 30°C for 2 d, YPD at 37°C for 2 d, and YPD plus 8 µg/mL fluconazole (FLC) at 30°C for 4 d. Multiplex PCR detect an extra Chr 13 in all of the strains with the fluconazole sensitivity observed in MN35. (B) The MN55 (n+19) and 20 isolates were spotted in 10-fold serial dilutions and incubated under the same conditions. Multiplex PCR confirmed that the isolates with the fluconazole resistant phenotype carry an extra chromosome 9. (TIF) [file pbio.1001653.s010.tif]

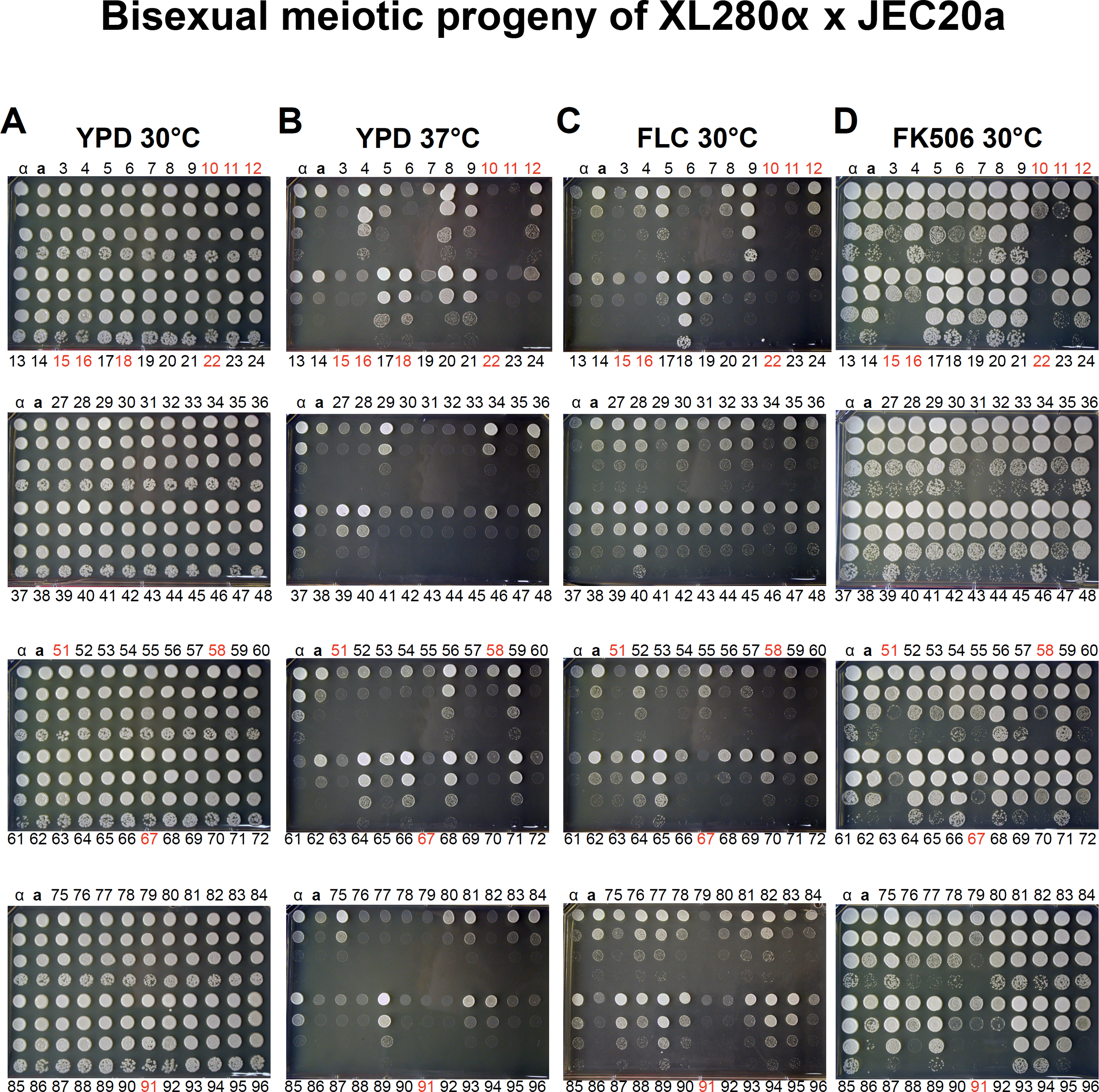

Supplement: Figure S11 — Opposite sexual reproduction progeny of XL280 crossed with JEC20 exhibit phenotypic variation. Strains were spotted in 10-fold serial dilutions and grown under the following conditions: (A) YPD at 30°C for 2 d, (B) YPD at 37°C for 2 d, (C) YPD plus 8 µg/mL fluconazole (FLC) at 30°C for 4 d, and (D) YPD plus 1 µg/mL FK506 at 30°C for 2 d. α and a represent the parental strains XL280 and JEC20, respectively. Red labels indicate the progeny whose phenotypes differed compared to the parental strains. (TIF) [file pbio.1001653.s011.tif]

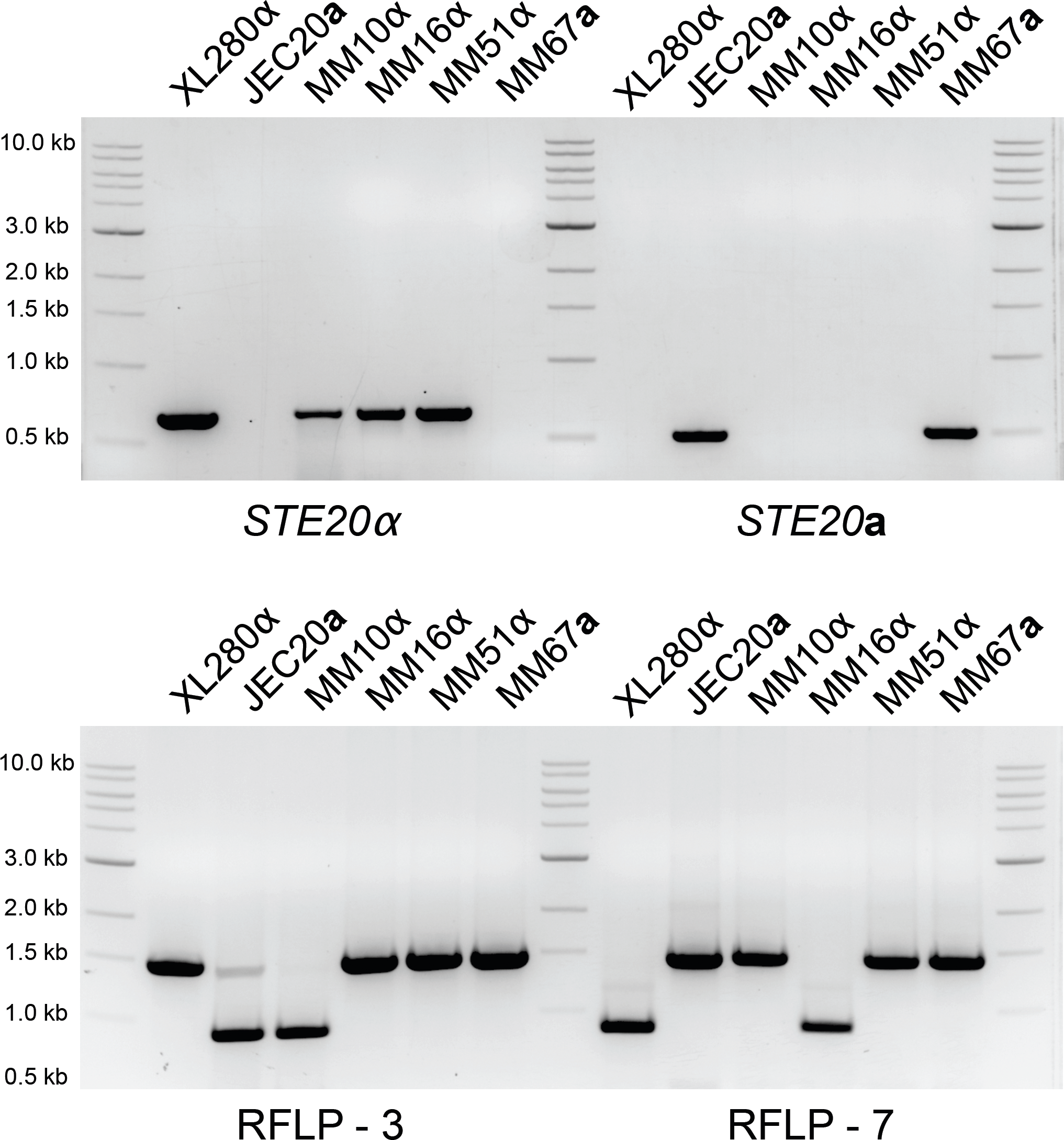

Supplement: Figure S12 — Aneuploid strains from the cross between XL280 and JEC20 are recombinants. Four markers were analyzed: STE20α, STE20 a, RFLP3, and RFLP7. MM10, MM16, MM51, and MM67 are four aneuploid progeny from the cross between strains XL280 and JEC20. (TIF) [file pbio.1001653.s012.tif]

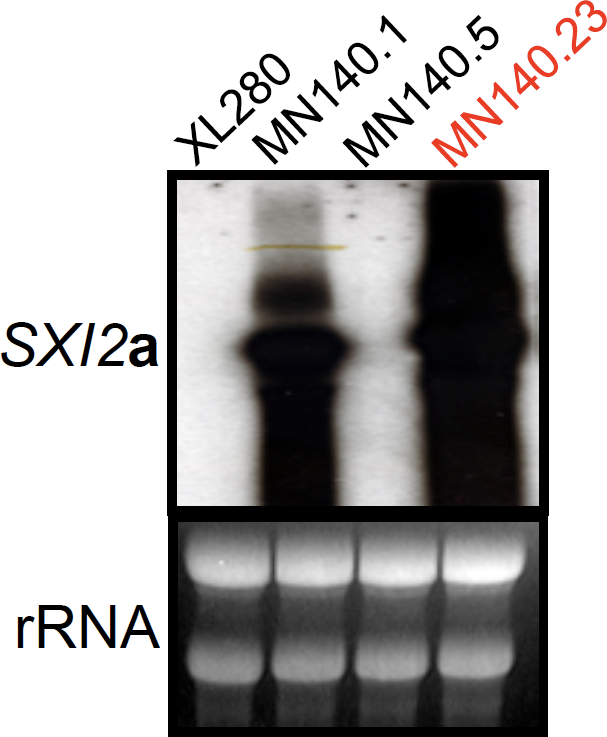

Supplement: Figure S13 — SXI2 a is overexpressed in MN140.23. Northern blot analysis using the SXI2 a gene as a probe detected the overexpressed SXI2 a gene in strain MN140.23, which contains a PGPD1::SXI2 a transgene in the XL280 background. EtBr staining of rRNA served as a loading control. (TIF) [file pbio.1001653.s013.tif]

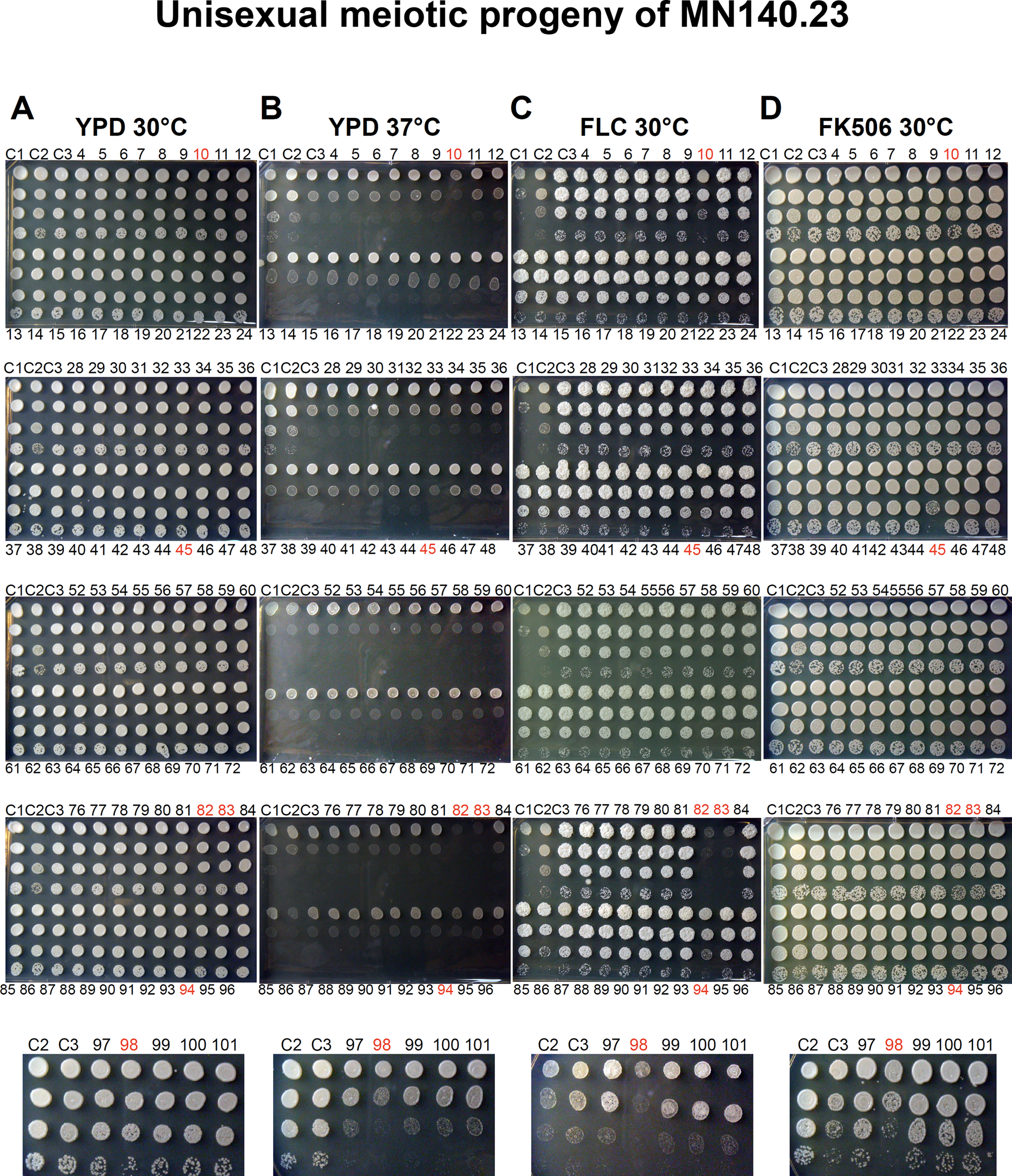

Supplement: Figure S14 — Unisexual reproduction progeny of MN140.23 (XL280, P GPD1 :: SXI2 a) exhibit phenotypic variation. Strains were spotted in 10-fold serial dilutions and grown under the following conditions: (A) YPD at 30°C for 2 d, (B) YPD at 37°C for 2 d, (C) YPD plus 8 µg/mL fluconazole (FLC) at 30°C for 4 d, and (D) YPD plus 1 µg/mL FK506 at 30°C for 2 d. C1, C2, and C3 represent strains XL280, XL566 (XL280 ura5), and MN140.23. Red labels indicate the progeny with phenotypes that differed compared to the parental strain. (TIF) [file pbio.1001653.s014.tif]

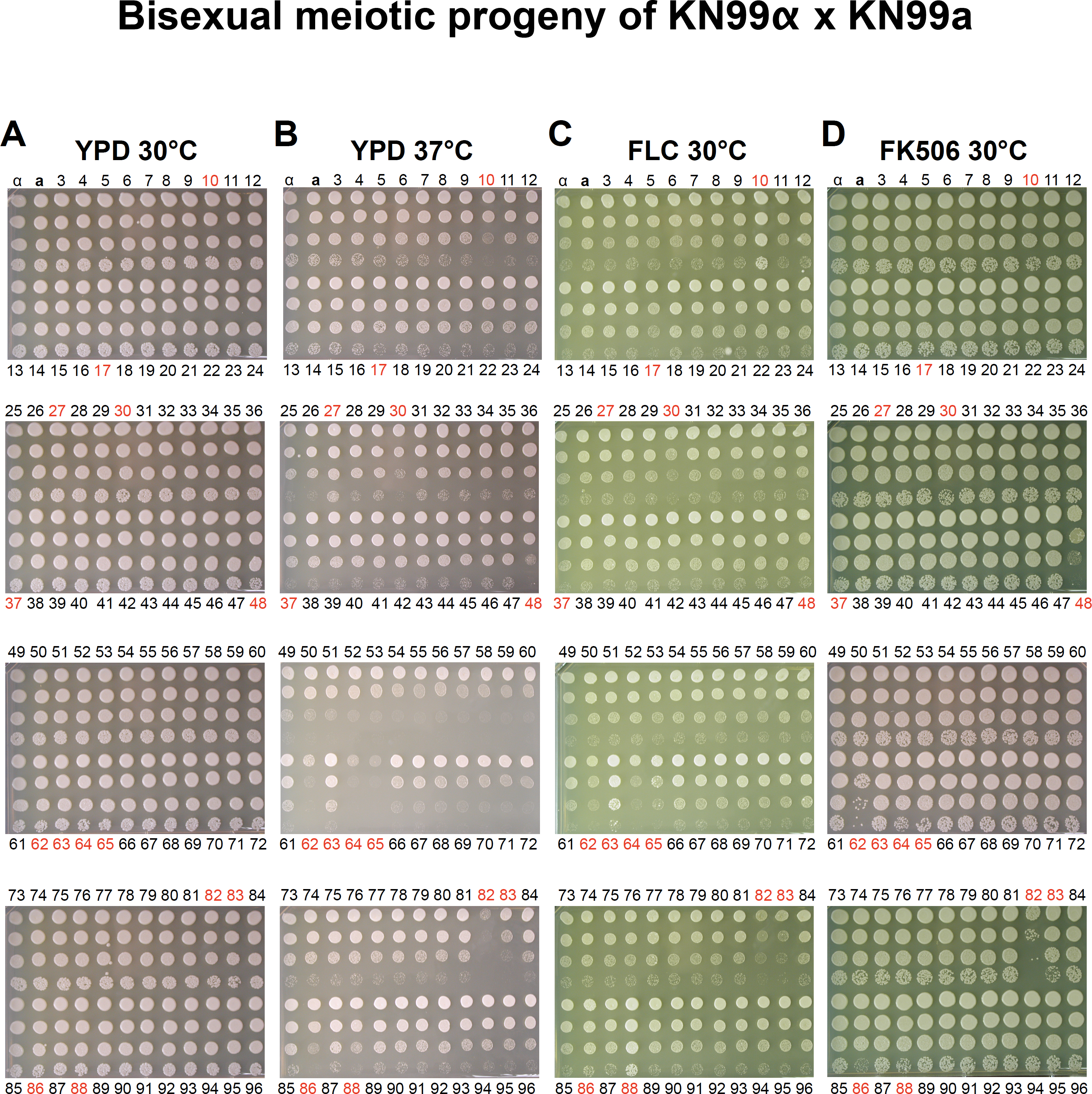

Supplement: Figure S15 — Opposite sexual reproduction progeny of isogenic serotype A strains KN99a and KN99α exhibit phenotypic variation. Strains were 10-fold serially diluted, spotted, and grown under the following conditions: (A) YPD at 30°C for 2 d, (B) YPD at 37°C for 2 d, (C) YPD plus 8 µg/mL fluconazole (FLC) at 30°C for 4 d, and (D) YPD plus 1 µg/mL FK506 at 30°C for 2 d. The progeny that phenotypically differed from the wild-type is marked red. (TIF) [file pbio.1001653.s015.tif]
