# Supplementary material for: Unisexual and Heterosexual Meiotic Reproduction Generate Aneuploidy and Phenotypic Diversity De Novo in the Yeast Cryptococcus neoformans
Source: PLoS Biol. 2013 Sep 10;11(9):e1001653. doi: 10.1371/journal.pbio.1001653 (PMC3769227; doi:10.1371/journal.pbio.1001653)
Supplement: Table S1 — Summary of reads from Illumina sequencing. (DOC) [file pbio.1001653.s016.doc]

**Table S1. Summary of reads from I**llumina sequencing

|  | **XL280** | **MN7** | **MN55** | **MN89** |
| --- | --- | --- | --- | --- |
| **Reads (each direction)** | 23,945,651 | 24,322,401 | 23,044,981 | 22,325,390 |
| **Total reads** | 47,891,302 | 48,644,802 | 46,089,962 | 44,650,780 |
|  |  |  |  |  |
| **Aligned to reference** | 43,355,876 | 42,496,619 | 39,373,747 | 40,734,202 |
| **Percentage (%)** | 90.5 | 87.4 | 85.4 | 91.2 |
