# Supplementary material for: Unisexual and Heterosexual Meiotic Reproduction Generate Aneuploidy and Phenotypic Diversity De Novo in the Yeast Cryptococcus neoformans
Source: PLoS Biol. 2013 Sep 10;11(9):e1001653. doi: 10.1371/journal.pbio.1001653 (PMC3769227; doi:10.1371/journal.pbio.1001653)
Supplement: Table S3 — Primers used in the multiplex PCR assay. (DOC) [file pbio.1001653.s018.doc]

**Table S3. Primers used in the multiplex PCR assay.**

| **Primer** | **Sequence (5’ to 3’)** | **Chr** | **Positions** | **Amplicon length (bp)** |
| --- | --- | --- | --- | --- |
| JOHE21844/MN174 | ACGTTTCTTGCCCTACATGG | 1 | 10514 | 131 |
| JOHE21845/MN175 | GTTGTTAGCACGGCCTTTTC | 1 | 10644 |  |
| JOHE21846/MN176 | GTTGCTGACACCTTGGAACA | 2 | 10778 | 192 |
| JOHE21847/MN177 | ACGTGTATAGGGCCAAGCAG | 2 | 10969 |  |
| JOHE21848//MN178 | ATCTATCGGAGTGCCTGGTG | 3 | 10027 | 306 |
| JOHE21849//MN179 | TAACCTACGCTTGGCGCTAC | 3 | 10332 |  |
| JOHE21850/MN180 | GGCGTCAAGTTCTCTTCTGG | 4 | 10335 | 405 |
| JOHE21851/MN181 | GCTGGACCTTTGTGGTTCAT | 4 | 10739 |  |
| JOHE21852/MN182 | GCTTTCGATGCCCATATTTC | 5 | 20031 | 491 |
| JOHE21853/MN183 | TTAGTTGCTCCGGTGTTTGA | 5 | 20521 |  |
| JOHE21854/MN184 | AAAGGTCCTCGAGTGTCACG | 6 | 10092 | 607 |
| JOHE21855/MN185 | TCCTTCAACCTCTCCCTTGA | 6 | 10698 |  |
| JOHE21856/MN186 | CGCCGAAGCGAGATTATTTA | 7 | 10298 | 701 |
| JOHE21857/MN187 | AAGGTGACCAAAGCACCAAC | 7 | 10998 |  |
| JOHE21858/MN188 | TACAAGCCGAGATTGCACAC | 8 | 80215 | 794 |
| JOHE21859/MN189 | AACTTTGGTAGCGGGTGATG | 8 | 81008 |  |
| JOHE21860/MN190 | AGGATTCCAAACCGGAGAGT | 9 | 10278 | 892 |
| JOHE21861/MN191 | CGATCCCTTCATGTCACCTT | 9 | 11169 |  |
| JOHE21862/MN192 | GACGTACGTGGCCTTGAAGT | 10 | 10123 | 1004 |
| JOHE21863/MN193 | CCGATTACCGACATGCCTAT | 10 | 11126 |  |
| JOHE21864/MN194 | CCACAGTCCCTGGTCATTCT | 11 | 10103 | 1103 |
| JOHE21865/MN195 | TGCCATCCATGACGATAGTG | 11 | 11205 |  |
| JOHE21866/MN196 | CTCTTTCAAACCGCCAAAAG | 12 | 90284 | 1196 |
| JOHE21867/MN197 | TGCTGAGGAAGCCTTGATTT | 12 | 91479 |  |
| JOHE21868/MN198 | GCAAGCGTCAATAACCCACT | 13 | 10166 | 1298 |
| JOHE21869/MN199 | GGCGTGTGAGTTCCATTCTT | 13 | 11463 |  |
| JOHE21870/MN200 | CCTCAGAATGCTGCTGACAA | 14 | 100243 | 1390 |
| JOHE21871/MN201 | ACACGGCGAAAAGGTTACAG | 14 | 101632 |  |
