# Supplementary material for: Unisexual and Heterosexual Meiotic Reproduction Generate Aneuploidy and Phenotypic Diversity De Novo in the Yeast Cryptococcus neoformans
Source: PLoS Biol. 2013 Sep 10;11(9):e1001653. doi: 10.1371/journal.pbio.1001653 (PMC3769227; doi:10.1371/journal.pbio.1001653)
Supplement: Table S4 — Strains and plasmids used in this study. (DOC) [file pbio.1001653.s019.doc]

**Table S4. Strains and plasmids used in this study.**

| Strain | | Genotype | Source/ Reference |
| --- | --- | --- | --- |
| *C. neoformans* var. *grubii* (serotype A) | | | |
| KN99**a** | | *MAT***a** | [68] |
| KN99α | | *MAT*α | [68] |
| MN503 to MN596 | | Mating progeny from the cross between KN99**a** and KN99α | This study |
| *C. neoformans* var. *neoformans* (serotype D) | | | |
| XL280 | | *MAT*α | [44] |
| JEC20**a** | | *MAT***a** | [46] |
| JEC21α | | *MAT*α | [46] |
| XL566 | | *MAT*α *ura5* | (Lin and Heitman, unpublished data) |
| MN1 to MN90 | | Progeny from same-sex mating of XL280 | This study |
| MN201 to MN290 | | Mating progeny from the **a**-α cross between JEC20**a** and JEC20α | This study |
| MN301 to MN390 | | XL280 mitotic progeny grown on YPD | This study |
| MN140.23 | | *MAT*α *ura5* P*_GPD1_*::*SXI2***a**::*URA5* | This study |
| MN401 to MN501 | | Progeny from α-α same-sex mating of MN140.23 | This study |
| MN711 to MN806 | | XL280 mitotic progeny grown on mating V8 media | This study |
| MF180 | | MN7 *NEO* | This study |
| XL561 | | XL280 *NAT* | (Lin and Heitman, unpublished data) |
| MF182 | | Diploid fusion isolate from the cross between MN7 *NEO* and XL280 *NAT* | This study |
| MF183 | | Diploid fusion isolate from the cross between MN7 *NEO* and XL280 *NAT* | This study |
| MF184 | | Diploid fusion isolate from the cross between MN7 *NEO* and XL280 *NAT* | This study |
| MF186 | | MN7 + pMF86 | This study |
| MF187 | | MN7 + pMF89 | This study |
| Plasmid | | | |
| pCH285 | P*_GPD1_*::*SXI2***a**::*URA5 Amp^R^* | | [54] |
| pMN7 | pCH285 without telomeric sites | | This study |
| pJAF12 | *NEO* *AMP* | | [63] |
| pMF86 | pJAF12 *HSC20 NEO AMP* | | This study |
| pMF89 | pJAF12 *HSC20 NEO AMP* | | This study |
