# Supplementary material for: Unisexual and Heterosexual Meiotic Reproduction Generate Aneuploidy and Phenotypic Diversity De Novo in the Yeast Cryptococcus neoformans
Source: PLoS Biol. 2013 Sep 10;11(9):e1001653. doi: 10.1371/journal.pbio.1001653 (PMC3769227; doi:10.1371/journal.pbio.1001653)
Supplement: Table S5 — Primers used in this study. (DOC) [file pbio.1001653.s020.doc]

**Table S5. Primers used in this study.**

| Primer | Sequence (5’ to 3’) | Comments |
| --- | --- | --- |
| JOHE9028 | ATGGGCAGCAACCTTGACATC | *SXI2***a** probe F |
| JOHE9870 | GGATAGATCTTACCCCCTGAGGACTGT | *SXI2***a** probe R |
| JOHE21313/WL | CACATCTCAGATGCCATTTTACCA | *STE20*_D**a**_F |
| JOHE21323/WL | TCATCACAATGATCTCATTCACAA | *STE20*_D**a**_R |
| JOHE21312/WL | AGCACCAGCCTATGGAGTCCGTCT | *STE20*_Dα_F |
| JOHE21322/WL | TCAAAAGGTTGTCAGACTTGATGT | *STE20*_Dα_R |
| JOHE24473/MN286 | CGAGGATCGTACAGTGCGTA | RFLP-3-NdeI F |
| JOHE24474/MN287 | CGCACTTCTTTCGTCATTCA | RFLP-3-NdeI R |
| JOHE24481/MN294 | TATTCCCTCCTTGCTTGGTG | RFLP-7-XbaI F |
| JOHE24482/MN295 | AACAACCACGTTTCCAGGTC | RFLP-7-XbaI R |
| JOHE24111/MN264 | AAGAGAGGGGGACGAACAAT | Hsc20 SNP_F |
| JOHE24112/MN265 | GCCGAGATTGTGTTGGATTT | Hsc20 SNP_R |
| JOHE38840/MF281 | GGTACCGGATTTATGGTGTAGATGAATG | Hsc20-KpnI_F |
| JOHE38841/MF282 | GGTACCAAGGACATCGGATAGAGATTA | Hsc20-KpnI_R |
| M13F | GTAAAACGACGGCCAG |  |
| M13R | CAGGAAACAGCTATGAC |  |
